# Highly active postspinel-structured catalysts for oxygen evolution reaction†

**DOI:** 10.1039/d2ra00448h

**Published:** 2022-02-10

**Authors:** Yuichi Okazaki, Seiji Oda, Akihiko Takamatsu, Shogo Kawaguchi, Hirofumi Tsukasaki, Shigeo Mori, Shunsuke Yagi, Hidekazu Ikeno, Ikuya Yamada

**Affiliations:** Department of Materials Science, Graduate School of Engineering, Osaka Prefecture University 1-1 Gakuen-cho, Naka-ku, Sakai Osaka 599-8531 Japan yamada@mtr.osakafu-u.ac.jp ikeno@mtr.osakafu-u.ac.jp; Department of Molecular Engineering, Graduate School of Engineering, Kyoto University Kyoto Daigaku Katsura, Saikyo-ku Kyoto 615-8510 Japan; Japan Synchrotron Radiation Research Institute (JASRI) 1-1-1 Kouto, Sayo-cho, Sayo-gun Hyogo 679-5198 Japan; Institute of Industrial Science, The University of Tokyo 4-6-1 Komaba, Meguro-ku Tokyo 153-8505 Japan; Precursory Research for Embryonic Science and Technology (PRESTO), Japan Science and Technology Agency (JST) 4-1-8 Honcho Kawaguchi Saitama 332-0012 Japan

## Abstract

The rational design principle of highly active catalysts for the oxygen evolution reaction (OER) is desired because of its versatility for energy-conversion applications. Postspinel-structured oxides, Ca*B*_2_O_4_ (*B* = Cr^3+^, Mn^3+^, and Fe^3+^), have exhibited higher OER activities than nominally isoelectronic conventional counterparts of perovskite oxides La*B*O_3_ and spinel oxides Zn*B*_2_O_4_. Electrochemical impedance spectroscopy reveals that the higher OER activities for Ca*B*_2_O_4_ series are attributed to the lower charge-transfer resistances. A density-functional-theory calculation proposes a novel mechanism associated with lattice oxygen pairing with adsorbed oxygen, demonstrating the lowest theoretical OER overpotential than other mechanisms examined in this study. This finding proposes a structure-driven design of electrocatalysts associated with a novel OER mechanism.

## Abstract 2

Postspinel-structured oxides, Ca*B*_2_O_4_ (*B* = Cr^3+^, Mn^3+^, and Fe^3+^), have exhibited systematically higher catalytic activities in the oxygen evolution reaction (OER) than nominally conventional counterparts of perovskite La*B*O_3_ and spinel Zn*B*_2_O_4_.

## Graphical abstract

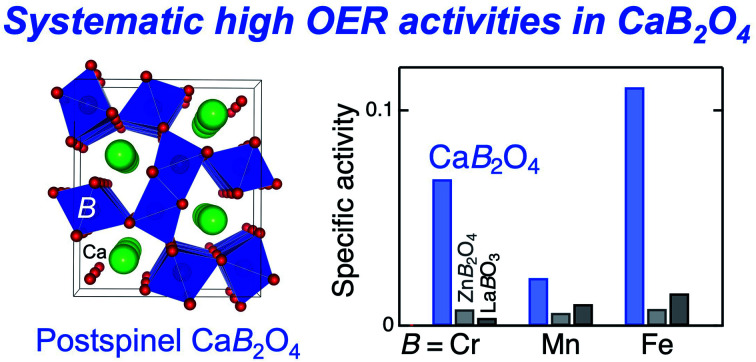

## Introduction

The oxygen evolution reaction (OER: 4OH^−^ → O_2_ + 2H_2_O + 4e^−^ in alkaline conditions) plays an essential role in energy-conversion applications such as water electrolysis and rechargeable metal–air batteries.^1–3^ Since this reaction intrinsically involves large overpotentials causing colossal energy loss, precious-metal oxides (*e.g.*, RuO_2_ and IrO_2_) are presently utilized as typical OER catalysts.^4–6^ Despite their high performance, large-scale applications are restricted because of their scarcity and high cost. Accordingly, much effort has been directed toward the development of highly active transition metal oxide catalysts consisting of earth-abundant and low-cost elements.^7,8^ Most of the transition metal oxide catalysts, such as spinel and perovskite, comprise tetrahedral and octahedral metal–oxygen units.^1,9–11^ The perovskite-structured oxides, one of the most well-studied experimentally and theoretically catalyst systems,^1,12^ consist of vertex-sharing octahedra, in which a single-site adsorption/reaction mechanism is widely accepted as adsorbates evolution reaction (AEM).^12,13^ The reactants are adsorbed on coordinatively unsaturated sites (CUS) formed by the extraction of oxygen at an octahedral vertex. Since the neighboring transition metal sites in vertex-sharing octahedra are far from each other, the bridging adsorption of adsorbates on two active sites is disturbed.

Several structures in transition metal oxides possess particular geometric conditions such as smaller transition-metal interatomic distances than that of vertex-sharing octahedra in perovskite, inducing interactions between adsorbates and multiple sites on the catalyst surface. Accordingly, dual-site adsorption/reaction mechanisms that the reactant bound to the CUS is also connected to another atom in the surrounding polyhedra are manifested by experiments and theoretical calculations.^5,14–17^ For example, the dual-site reaction mechanism bridging *B*-site octahedral CUS metal and *A*′-site pseudo-square coordinated transition metal has been suggested in the *A*-site-ordered quadruple perovskite (*AA*′_3_*B*_4_O_12_).^15,16^ The dual-site reaction mechanism has been experimentally and theoretically examined in the rutile-structured RuO_2_.^5,18^ RuO_2_ is composed of one-dimensional edge-shared RuO_6_ octahedral chains gathered by sharing vertices. The reaction mechanism reported in the RuO_2_(110) surface involves a reaction step where an oxygen atom adsorbed on the Ru CUS combines with the oxygen atom (O_BRI_) bridging two Ru atoms in the octahedral chain neighboring to CUS.^5^ Recently, Sugawara *et al.* reported that CaFe_2_O_4_ exhibits higher OER activity than other Fe oxides,^17^ suggesting a novel reaction mechanism in which three Fe atoms participate in direct O–O bond formation based on density-functional-theory (DFT) calculations. Although the geometric feature associated with multi-site adsorption and reaction is reasonably described, the unusual 3-step reaction *via* simultaneous adsorption of two OH^−^ species on Fe CUSs (Fe_CUS_) and two electrons transfer is assumed in this mechanism, in contrast to the ordinary 4-step reaction in which one OH^−^ and one electron are sequentially involved at each step.

**Fig. 1:**
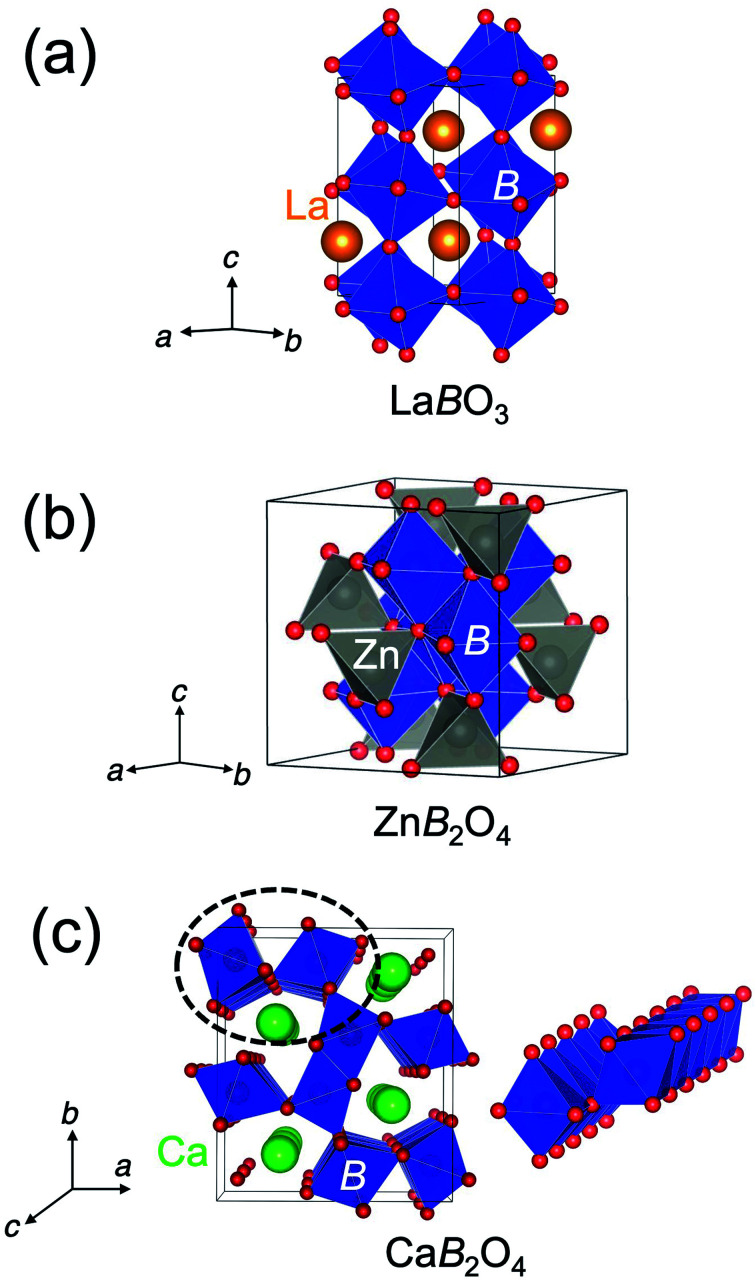
Schematics of crystal structures of spinel (a) La*B*O_3_, (b) Zn*B*_2_O_4_, and (c) postspinel-structured Ca*B*_2_O_4_ (CaFe_2_O_4_-type). The one-dimensional edge-sharing *B*O_6_ octahedral structure in the dashed circle is illustrated on the right.

The crystal structure of CaFe_2_O_4_ consists of edge-sharing FeO_6_ octahedra chains like rutile. It can be classified as the postspinel structure, a high-pressure polymorph of spinel. Fig. 1 shows the crystal structures of perovskites, spinels, and postspinels. CaFe_2_O_4_-type postspinel structure has the one-dimensional framework of octahedra with shared edges, including Ca ions in the voids, distinct from spinel and perovskite with the three-dimensional octahedral framework with shared edges and corners, respectively. Considering that the OER activity of CaFe_2_O_4_ may be derived from the structural feature, the postspinel-related series of Ca*B*_2_O_4_ (*B* = Cr and Mn) must exhibit higher OER catalytic activity than the spinel or perovskite oxides.

In this paper, we investigated the OER catalytic activities of postspinel-structured Ca*B*_2_O_4_ (*B* = Cr, Mn, and Fe) and systematically compared activities with perovskite La*B*O_3_ and spinel Zn*B*_2_O_4_. Regardless of *B*-site transition metals, the OER activities in Ca*B*_2_O_4_ oxides are monotonically superior to those of Zn*B*_2_O_4_ and La*B*O_3_ counterparts, which is supported by lower charge-transfer resistance in Ca*B*_2_O_4_. We performed DFT calculations to reveal the origin of OER activity in CaFe_2_O_4_ by remodeling the regular 4-step reaction mechanism from the previously reported 3-step mechanism^17^ and compared with the comparison with several possible mechanisms. We eventually found a novel 4-step reaction mechanism with lower theoretical overpotential, where the adsorbed oxygen on the Fe_CUS_ and the adjacent O_BRI_ were desorbed to generate oxygen. This finding suggests a new design principle for improving catalytic activity in multiple crystal structures of transition metal oxides.

## Experimental

### Material synthesis

CaCr_2_O_4_, CaMn_2_O_4_, and CaFe_2_O_4_ were obtained from the mixtures of CaCO_3_ (99.95%) and Cr_2_O_3_ (99.9%), MnO_2_ (99.99%), or Fe_2_O_3_ (99.99%) by calcining at 1473, 1473, and 1373 K, respectively, for 10–24 h for several times. ZnCr_2_O_4_ and ZnMn_2_O_4_ were synthesized from the mixtures of ZnO (99.9%) and Cr_2_O_3_ (99.9%) or MnO_2_ (99.9%) by heating at 1273 and 1173 K for 5 and 10 h, respectively. ZnFe_2_O_4_ was obtained using the polymerized complex method.^19^ A mixture of ZnO (99.9%) and Fe(NO_3_)_3_·9H_2_O (99.9%) at a molar ratio of 1 : 2 was dissolved in nitric acid solution (∼5 M), to which a five-fold excess of citric acid and one-fold excess of 1,2-ethanediol were added to the solution with stirring. The resulting solution was heated for 573 K and maintained at this temperature for 1 h to dry. Subsequently, the dried powder was fired using a furnace at 673 K for 1 h and then 1273 K for 10 h in air with occasional grindings. LaCrO_3_ and LaMnO_3_ were also obtained using the polymerized complex method from mixtures of La(NO_3_)_3_·6H_2_O and Cr(NO_3_)_3_·9H_2_O or La(NO_3_)_3_·6H_2_O and Mn(NO_3_)_3_·6H_2_O, by combustion at 1273 and 1073 K for 5 and 10 h, respectively. LaFeO_3_ was synthesized from a stoichiometric mixture of La_2_O_3_ and Fe_2_O_3_ by heating at 1673 K for 10 h.

### Basic characterization

The as-synthesized samples were identified by X-ray powder diffraction (XRD) with Cu-Kα radiation (Ultima IV, Rigaku, Japan). The synchrotron XRD (SXRD) patterns were collected using a Debye–Scherrer camera installed at the BL02B2 beamline in SPring-8, Japan. The wavelength was determined as 0.49968 Å using CeO_2_ as a reference. The SXRD data were analyzed using the Rietveld refinement program RIETAN-FP.^20^ Specific surface areas were determined by Brunauer–Emmett–Teller (BET) analysis of Kr gas adsorption data (BELSORP-max, MicrotracBEL, Japan). The morphologies of all the catalysts were confirmed by scanning electron microscopy (SEM) images (TM3030, Hitachi High-Tech, Japan). X-ray absorption near edge structure (XANES) spectra of Cr, Mn, and Fe K-edges were collected in the transmission mode at the BL14B2 beamline in SPring-8. The X-ray absorption spectra were normalized by spline functions between pre-edge and post-edge regions using Athena program of the IFEFFIT package.^21^

### Electrochemical characterization

Working electrodes were prepared using the drop-casting method of inks containing catalysts on glassy carbon electrode, referred to previous papers.^10^ A 5 wt% proton-type Nafion suspension (Sigma-Aldrich), 0.1 M KOH aqueous solution (Nacalai Tesque, Inc., Japan), and tetrahydrofuran (THF, Sigma-Aldrich) were mixed at a ratio of 2 : 1 : 97 in volume. The catalyst ink was prepared by mixing 5 mg of catalyst, 1 mg of acetylene black (Denka Co., Ltd, Japan), and 1 mg of the THF solution. A 6.4 μL of catalyst ink was taken with stirring and drop cast onto the glassy-carbon disk electrode with 4 mm diameter.

Electrochemical measurements were conducted using a rotating-disk electrode rotator (RRDE-3 A, BAS Inc., Japan) and a bipotentiostat (model-2325, BAS Inc., Japan). We used a Pt wire electrode and a Hg/HgO electrode (International Chemistry Co., Ltd, Japan) filled with a 0.1 M KOH aqueous solution (Nacalai Tesque, Inc., Japan) as the counter and reference electrodes, respectively. All electrochemical measurements were conducted under O_2_ saturation at room temperature. This fixed the equilibrium potential of the O_2_/H_2_O redox couple to 0.304 V *versus* (*vs.*) Hg/HgO. The disk potential was controlled between 0.3 and 0.9 V *vs.* Hg/HgO at a scan rate of 10 mV s^−1^. The disk potential was represented in those *vs.* reversible hydrogen electrode (RHE), with *IR*-compensation (*R* = 43 Ω). The capacitive effect was compensated by averaging the cathodic and anodic scans.

Chronoamperometry (CA) was conducted at 1.6 V *vs.* RHE, where *IR*-compensation was not made. The electrochemical surface area (ECSA) was determined by scanning non-faradaic region between 0.0 and 0.1 V *vs.* Hg/HgO, according to the literature.^22^ Electrochemical impedance spectroscopy (EIS) measurement was conducted using an electrochemical analyzer (760E, BAS Inc., Japan) at 1.7 V *vs.* RHE at frequencies ranging from 0.1 Hz to 1 MHz.

## Density-functional-theory calculation

### Bulk model electronic structure

Spin-polarized DFT calculations were systematically performed for spinel and postspinel oxides, namely, CaFe_2_O_4_, CaCr_2_O_4_, CaMn_2_O_4_, ZnCr_2_O_4_, ZnMn_2_O_4_, and ZnFe_2_O_4_, using the plane-wave based projector augmented wave (PAW) method as implemented in the Vienna *ab initio* Simulation Package (VASP).^23–25^ The generalized gradient approximation (GGA) parametrized by Perdew, Burke, and Ernzerhof (PBE)^26^ were adopted to express exchange–correlation interactions. The strong on-site coulombic interactions on the localized 3d electrons were treated with the GGA + *U* approach.^27^ The *U*_eff_ = 3.5, 4.0, and 3.9 eV were adopted for Cr, Mn, and Fe 3d orbitals, which were selected to reproduce the experimental oxidation enthalpy, as reported previously.^28,29^ The PAW potential data-set with radial cutoffs of 2.3 Å for Ca, Cr, Mn, Fe, Zn, and 1.52 Å for O were employed, where Ca-3s, 3p, 4s, Cr-3p, 3d, 4s, Mn-3p, 3d, 4s, Fe-3d, 4s, Zn-4s, 4p, 3d, O-2s, 2p were described as valence electrons. Table S11† summarizes the magnetic structures and nominal electron configurations considered in this work. The plane-wave cutoff energy was set to 500 eV for all calculations. The Brillouin zone was sampled using *k*_1_ × *k*_2_ × *k*_3_ mesh points according to the Monkhorst–Pack scheme.^30^ The mesh count for each direction was selected as the near natural number of 35 per lattice parameter (1 Å^−1^). The lattice constants and internal coordinates were optimized until the total energy difference and residual forces converged to less than 10^−5^ eV and 10^−2^ eV Å^−1^, respectively. According to literature,^31–33^ oxygen 2p band centers and unoccupied 3d band centers of transition metal atoms were computed from the projected density of states (DOS) as follows:1
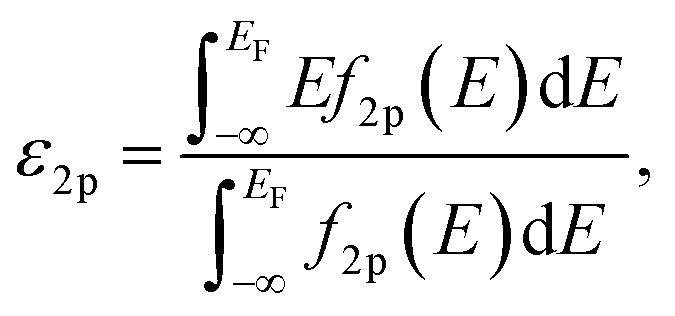
and2
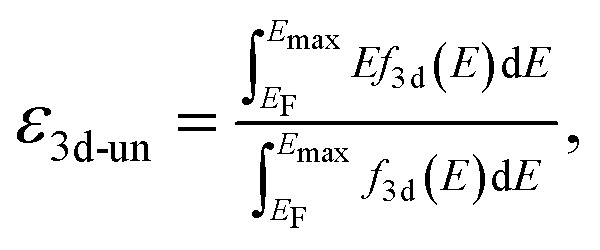
respectively. Here, *f*_2p_(*E*) and *f*_3d_(*E*) are DOS projected on O-2p and transition metal 3d orbitals, respectively; *E*_F_ is the Fermi energy; and *E*_max_ is the upper bound of unoccupied 3d bands. The *E*_max_ value was set as 10 eV higher than that of *E*_F_. The number of conduction bands was increased until the shapes of projected DOS were converged.

### Slab model surface energy and theoretical overpotential

The electronic structures of OER intermediates on the (001) surface of CaFe_2_O_4_ terminated by exposed FeO_5_ pyramids were investigated using DFT calculations. The slab models in Fig. S6† were composed of doubled cells along the *c* and *b* axes for CaFe_2_O_4_. The number of layers and the length of the vacuum layer in the slab models were carefully determined by checking the convergences of surface energies. For the (001) plane in CaFe_2_O_4_, the slab models respectively consisted of 116 atoms with 8 layers. The lattice constants of slab models for CaFe_2_O_4_ were 9.29 Å × 6.07 Å × 43.1 Å, including 20 Å vacuum layer to prevent interactions between surfaces in slab models. For these calculations, Brillouin zones were sampled with 4 × 6 × 1 grids for CaFe_2_O_4_. We fixed the positions of 81 atoms in the middle of these models for CaFe_2_O_4_ to evaluate bare surface energies (blue areas in Fig. S6b†). The atomic positions were optimized except for atomic layers in the bottom of slab models (magenta areas in Fig. S6b†) to calculate surface energies. The other computational conditions, including the PAW data-set, *U*_eff_ values, plane-wave cutoff energies, total energy differences, and residual forces, were identical with bulk calculations.

The surface energies of CaFe_2_O_4_ under the equilibrium conditions in OER were calculated according to the procedure proposed in the literature.^15,18^ The surface Gibbs free energy can be described for CaFe_2_O_4_ as follows:3
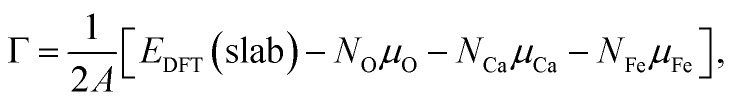
where *E*_DFT_(slab) is the total energy of the slab model using DFT calculations; *A* is the surface area of the slab model. *N*_Z_ and *μ*_Z_ (Z = Ca, Fe, and O) are defined as the number of the atoms in the slab model and chemical potentials, respectively. The chemical potentials are determined under the equilibrium condition of water splitting. In agreement with the computational hydrogen electrode model described in the literature,^34,35^ the chemical potential of oxygen can be expressed as a function of pH and *ϕ*, the potential difference between the working electrode and the reference electrode, as follows:

4 *μ*_O_(pH,*ϕ*) = [*E*_DFT_(H_2_O(g)) + [ZPE-*TS*]_H_2_O_] − [[*E*_DFT_(H_2_(g)] + [ZPE-*TS*]_H_2__] + 2(*k*_B_*T*ln *a*_H^+^_ − *eϕ*),

where *E*_DFT_(H_2_O(g)) and *E*_DFT_(H_2_(g)) are the total energies of H_2_O and H_2_ molecular, respectively; [ZPE-*TS*]_H_2_O_ and [ZPE-*TS*]_H_2__ are the zero-point energy (ZPE) correction and entropy contribution, respectively; *T* is temperature; *k*_B_ is the Boltzmann constant; and *a*_H^+^_ is the proton activity. The surface is regarded to be in equilibrium with the bulk CaFe_2_O_4_ phase. Then, the sum of the chemical potentials satisfies the following formula:

5 *μ*_Ca_ + 2*μ*_Fe_ + 4*μ*_O_(pH,*ϕ*) = *E*_DFT_(CaFe_2_O_4_)

where *E*_DFT_(CaFe_2_O_4_) is the total energy of bulk CaFe_2_O_4_. By solving eqn (3) for *μ*_Fe_ and substituting it with eqn (4) into (3), the surface energy *Γ* is obtained as a linear function dependent on *μ*_Ca_.

In this work, we constructed reaction mechanisms from reported AEM^12,13^ and lattice-oxygen-mediated mechanism (LOM)^13^ and conducted surface calculations for the mechanisms listed in Tables S12 and S13:† AEM–O_BRI_, the LOM–O_BRI_, AEM model, and dual-site AEM models referred by Sugawara *et al.*^17^ In these reaction steps, the *X/*Y surface state of postspinel-structured CaFe_2_O_4_ is determined as using the binding state *X for Fe_CUS_ and the binding state *Y for adjacent Fe_CUS_ with O_BRI_. The – bondings of *O–O_BRI_ and *OOH–O_BRI_ surfaces exhibit interactions between adsorbed oxygen and O_BRI_. For each of the individual surfaces, the free energy change ΔG_*X/*Y_ (*X/*Y: adsorbed surfaces) was calculated using equations in Table S14.† For each of the six reaction mechanisms, the free energy change Δ*G*_*n*_ (*n*: reaction steps) in the individual reaction was defined as each formula in Tables S15 and S16.† Using the largest Δ*G*_*n*_ (*n*: reaction step), the value of theoretical overpotential (*η*_th_) was calculated using the following equation:

6 *η*_th_ = max{Δ*G*_1_, Δ*G*_2_, Δ*G*_3_, Δ*G*_4_}/*e* − 1.23 [V].

## Results and discussion

**Fig. 2:**
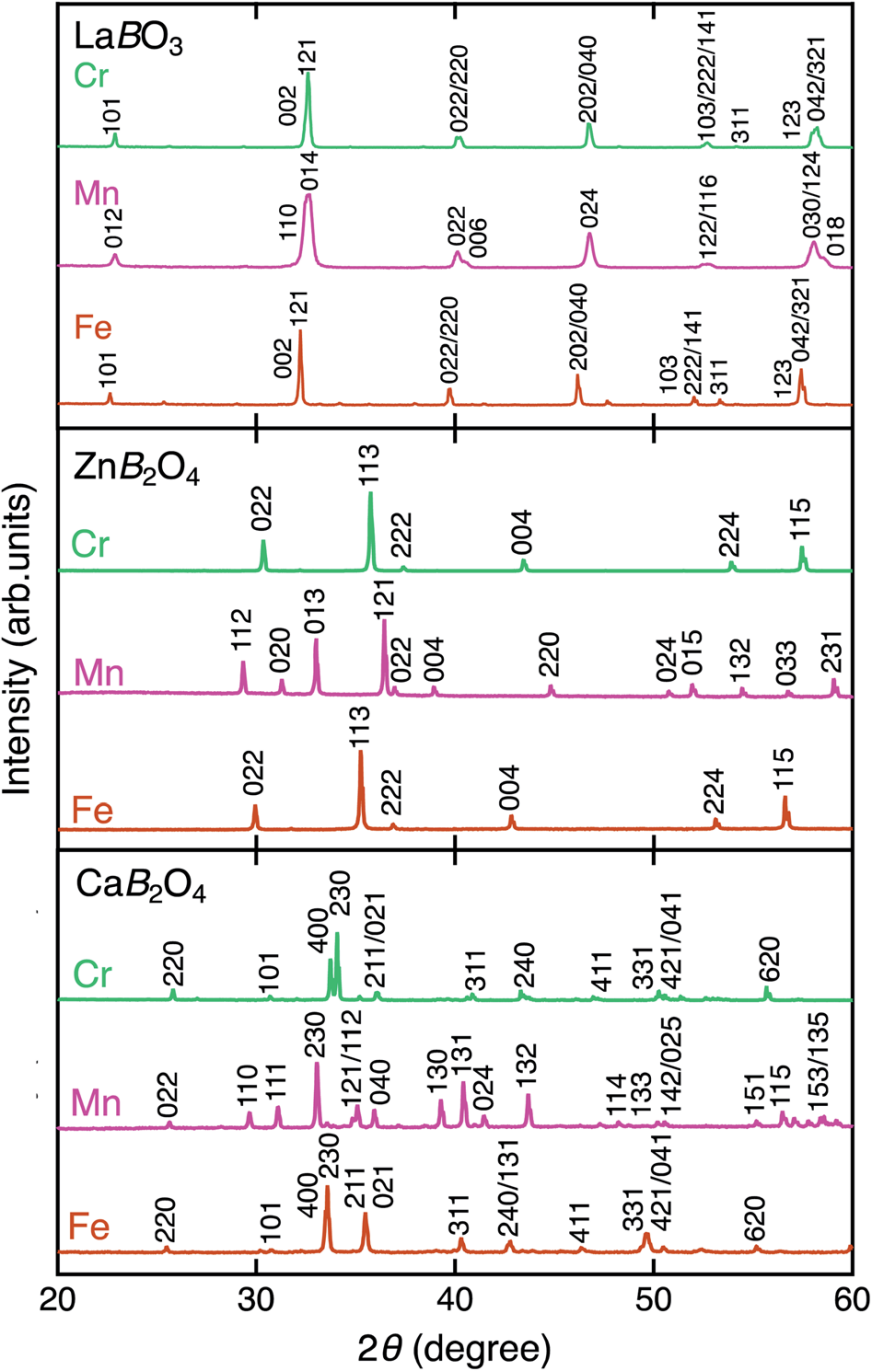
XRD patterns of La*B*O_3_, Zn*B*_2_O_4_, and Ca*B*_2_O_4_ for *B* = Cr (green), Mn (magenta), and Fe (brown).

**Fig. 3:**
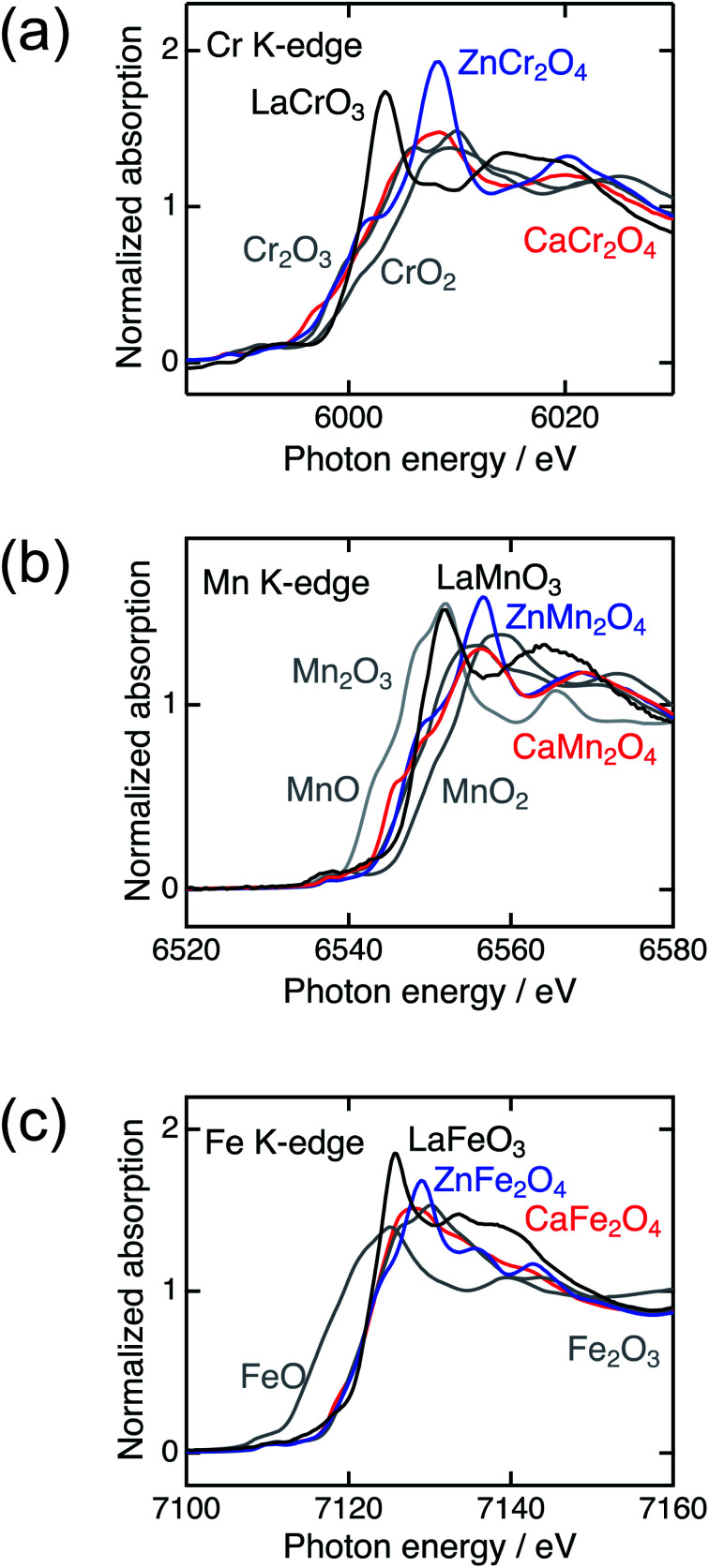
X-ray absorption spectra of transition metal K-edges for La*B*O_3_ (black), Zn*B*_2_O_4_ (blue), Ca*B*_2_O_4_ (red) (*B* = Cr, Mn, and Fe), and references (gray).

Fig. 2 shows the XRD patterns of La*B*O_3_, Zn*B*_2_O_4_, and Ca*B*_2_O_4_ (*B* = Cr, Mn, Fe). All samples crystallized in a single phase. Ca*B*_2_O_4_ were assigned to orthorhombic phases, as reported previously.^36–38^ Rietveld refinement results obtained by using the SXRD data confirmed that the refined lattice parameters were similar to those previously reported in all samples (Fig. S1 and Tables S1–S9†). The calculated bond valence sums (BVSs) indicate trivalent states of transition metal ions, as expected from the simple ionic models of La^3+^*B*^3+^O_3_^2−^, Zn^2+^*B*_2_^3+^O_4_^2−^, and Ca^2+^*B*_2_^3+^O_4_^2−^. Fig. 3 shows the X-ray absorption spectra at K-edges of Cr, Mn, and Fe. The K-edge absorption positions of transition metals for perovskites, spinels, and postspinels are close to those of pure trivalent metal oxide references (*B*_2_^3+^O_3_) rather than aliovalent references (*B*^2+^O and *B*^4+^O_2_), although the differences in local structures around the *B* sites appeared in shapes in higher energy ranges than absorption energies. The structural and spectroscopic analyses exclude the possible effects of valence on OER activities,^33^ thus the effects of crystal structure on activity can be investigated in this study.

Specific surface areas determined by the BET analysis of Kr-gas adsorption data ranged in typical values between ∼1.1 and ∼2.5 m^2^ g^−1^, as listed in Table 1. The BET specific surface areas were adopted to normalize the current densities per surface area of catalysts in the electrochemical analysis. The values of ECSA in Table 1 were observed in the region of 10–27 m^2^ g^−1^, displaying the same proportion of the maximum to the minimum, compared to that of BET surface areas. The SEM images in Fig. S2† indicate that the particle sizes roughly ranged between 0.1 and 10 μm for all. The crucial differences in grain size were not observed between Ca*B*_2_O_4_ and references containing the same *B* ion. The SEM observations roughly confirmed similar morphologies among all samples, compatible with BET and ECSA analyses.

**Table 1:** Specific surface area from BET analysis and electrochemical surface area (ECSA) for La*B*O_3_, Zn*B*_2_O_4_, and Ca*B*_2_O_4_ (*B* = Cr, Mn, Fe). Overpotential (*η*_0.05_), specific activity normalized by BET surface area at 1.6 V *vs.* RHE, and Tafel slope for these catalysts

Compound	Specific surface area (m^2^ g^−1^)	ECSA (m^2^ g^−1^)	*η*_0.05_ (V)	Specific activity (mA cm_oxide_^−2^)	Tafel slope (mV dec^−1^)
LaCrO_3_	2.49	27.3	0.75	0.004	230
LaMnO_3_	1.52	26.6	0.51	0.010	163
LaFeO_3_	1.32	20.5	0.42	0.015	78
ZnCr_2_O_4_	1.84	15.3	0.47	0.008	248
ZnMn_2_O_4_	1.57	21.3	0.48	0.005	101
ZnFe_2_O_4_	1.15	10.9	0.45	0.009	103
CaCr_2_O_4_	1.15	18.2	0.31	0.068	138
CaMn_2_O_4_	1.32	17.5	0.39	0.012	84
CaFe_2_O_4_	1.11	10.3	0.33	0.111	53

**Fig. 4:**
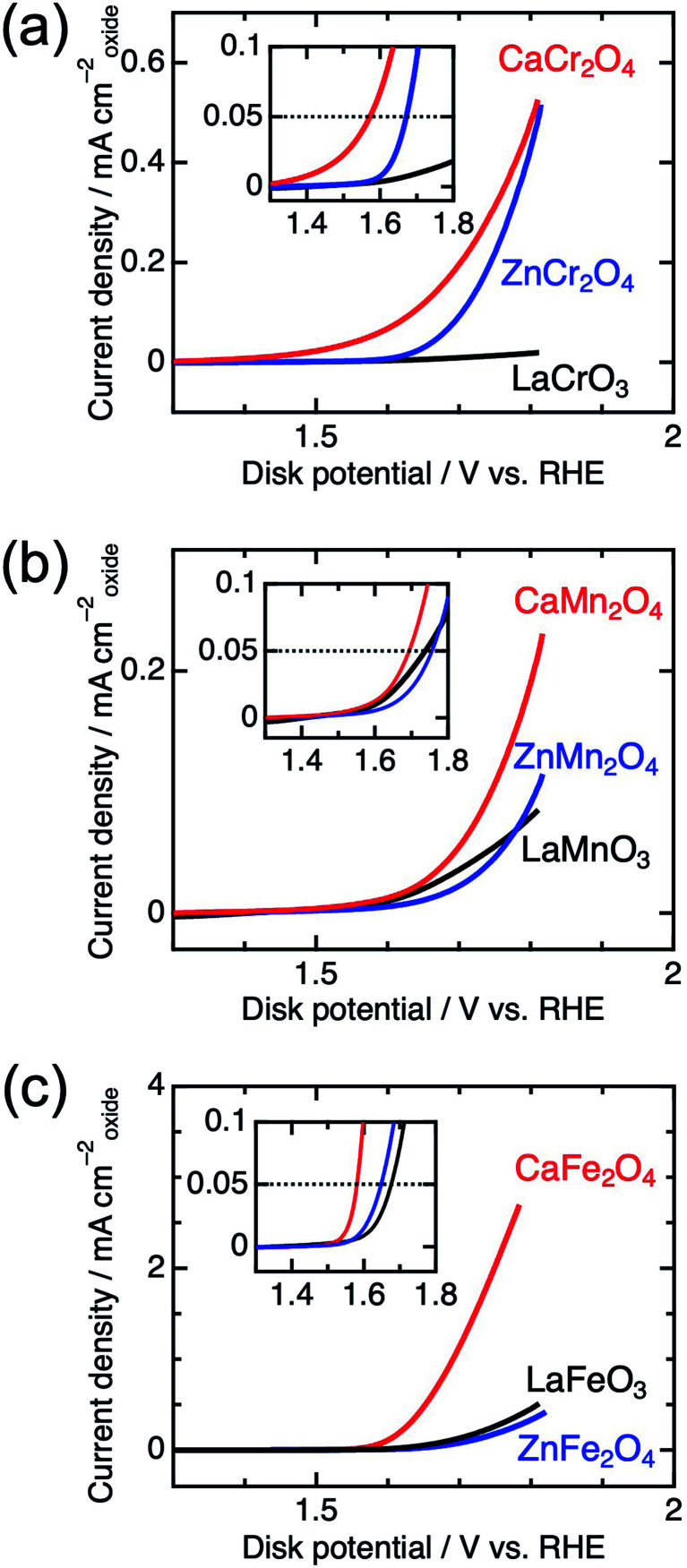
Linear sweep voltammograms for La*B*O_3_, Zn*B*_2_O_4_, and Ca*B*_2_O_4_ for *B* = (a) Cr, (b) Mn, and (c) Fe. The insets represent the magnified data in the vicinity of the OER onset potential.

**Fig. 5:**
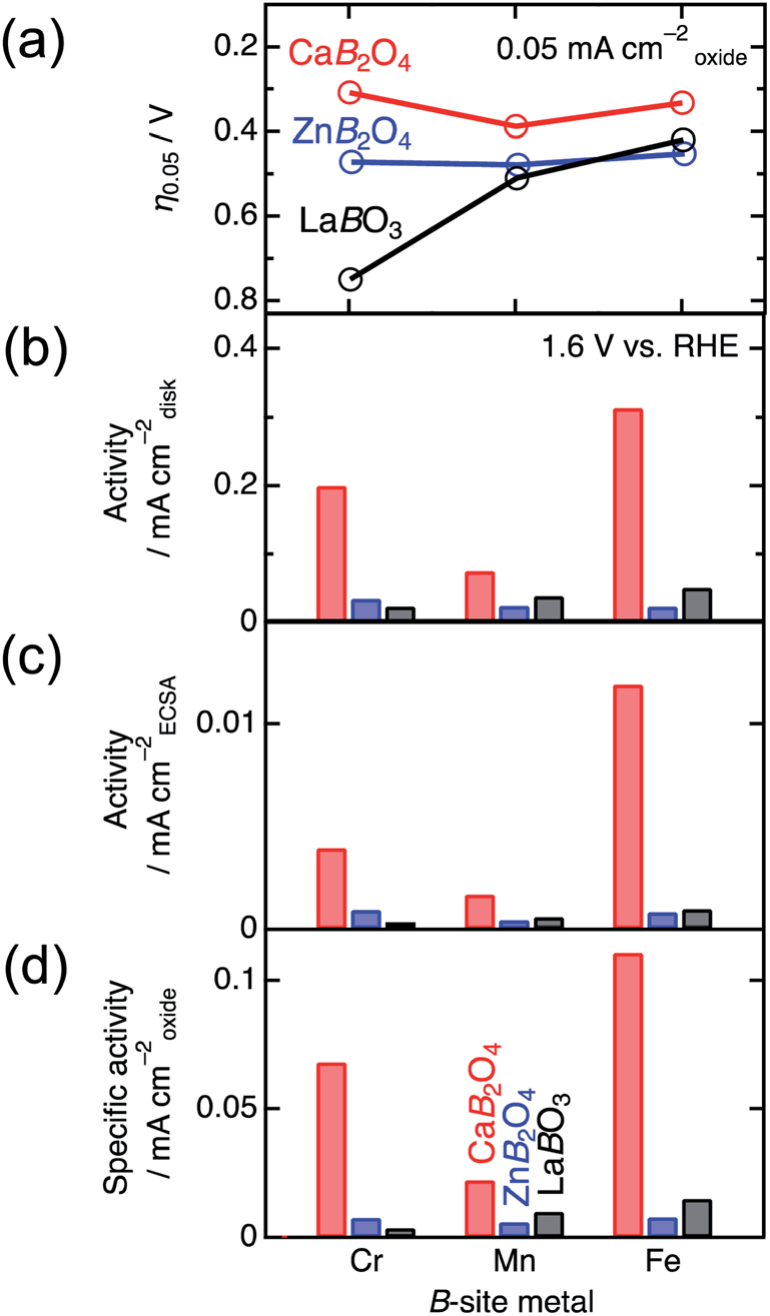
Comparison of OER (a) overpotential (*η*_0.05_) and activities at 1.6 V *vs.* RHE for La*B*O_3_, Zn*B*_2_O_4_, and Ca*B*_2_O_4_ (*B* = Cr, Mn, and Fe) normalized by (b) disk area (0.126 cm^−2^), (c) ECSA, and (d) BET surface area.

Fig. 4 shows the linear sweep voltammograms for La*B*O_3_, Zn*B*_2_O_4_, and Ca*B*_2_O_4_ (*B* = Cr, Mn, and Fe). Obviously, Ca*B*_2_O_4_ series exhibited much higher activities than the La*B*O_3_ and Zn*B*_2_O_4_ counterparts in the identical transition metal element. Taking Cr oxides for instance, the overpotential of CaCr_2_O_4_ was *η*_0.05_ = 0.34 V, substantially lower than those of ZnCr_2_O_4_ (0.44 V) and LaCrO_3_ (0.75 V) (see the inset of Fig. 4a and Table 1), where the overpotentials (*η*_0.05_) were determined at the onset potentials (*E*_onset_ V *vs.* RHE) exceeding the current density of 0.05 mA cm_oxide_^−2^: *η*_0.05_ = *E*_onset_ − 1.23 (V). The specific activity (a current density at 1.6 V *vs.* RHE) of CaCr_2_O_4_ was about 9 times higher than that of ZnCr_2_O_4_ or LaCrO_3_. Fig. 5 and Table 1 summarize the specific activities and overpotentials for all samples. The superiority of Ca*B*_2_O_4_ as OER catalyst were commonly observed in the activities normalized by disk areas, ECSA, and BET surface areas (Fig. 5). Regardless of the constituent transition metals, the lower overpotentials and larger activities reveal the intrinsic superiority of the postspinel structures. Especially, CaFe_2_O_4_ exhibited about 30 times larger specific activity and 0.15 V smaller *η*_0.05_ than ZnFe_2_O_4_. The improvement from spinel-structured ZnFe_2_O_4_ to postspinel-structured CaFe_2_O_4_ was the most remarkable among all comparisons.

**Fig. 6:**
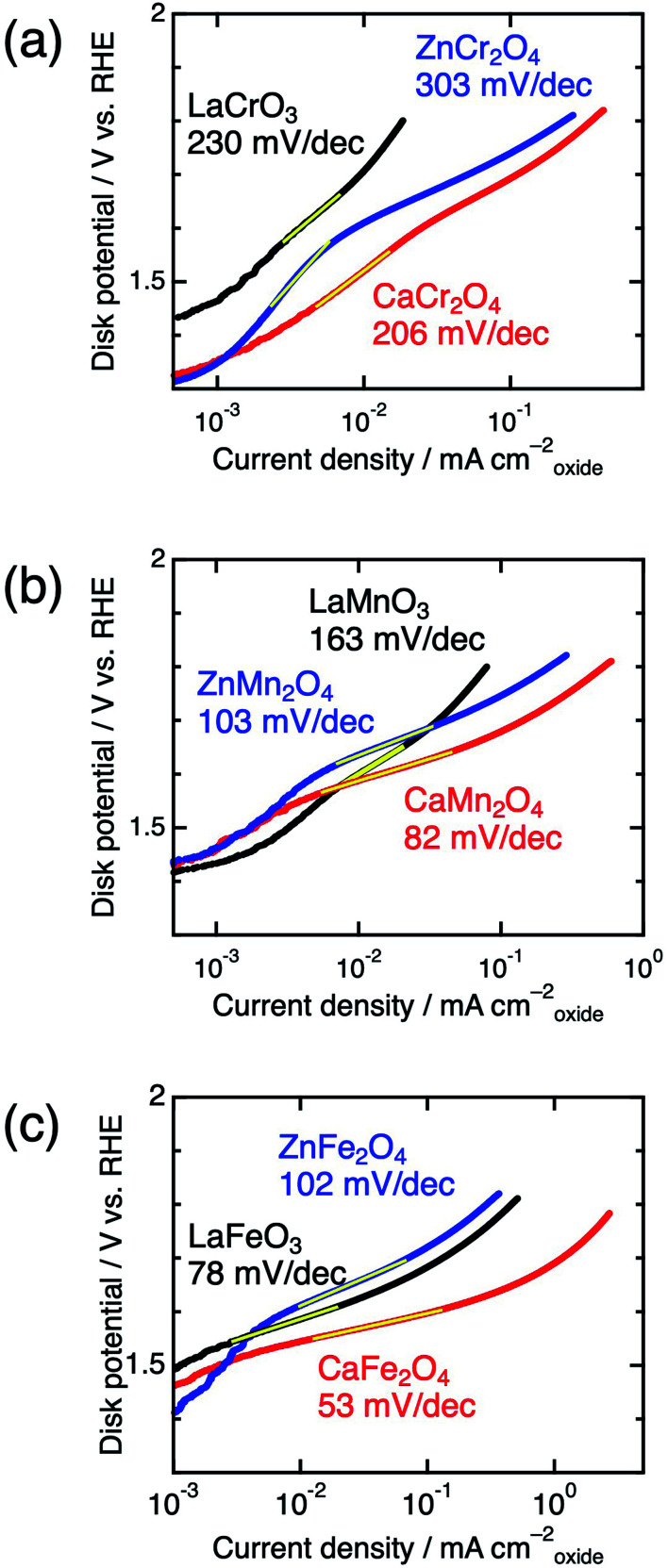
Tafel plots of La*B*O_3_ (black), Zn*B*_2_O_4_ (blue), and Ca*B*_2_O_4_ (red) for *B* = (a) Cr, (b) Mn, and (c) Fe.

**Fig. 7:**
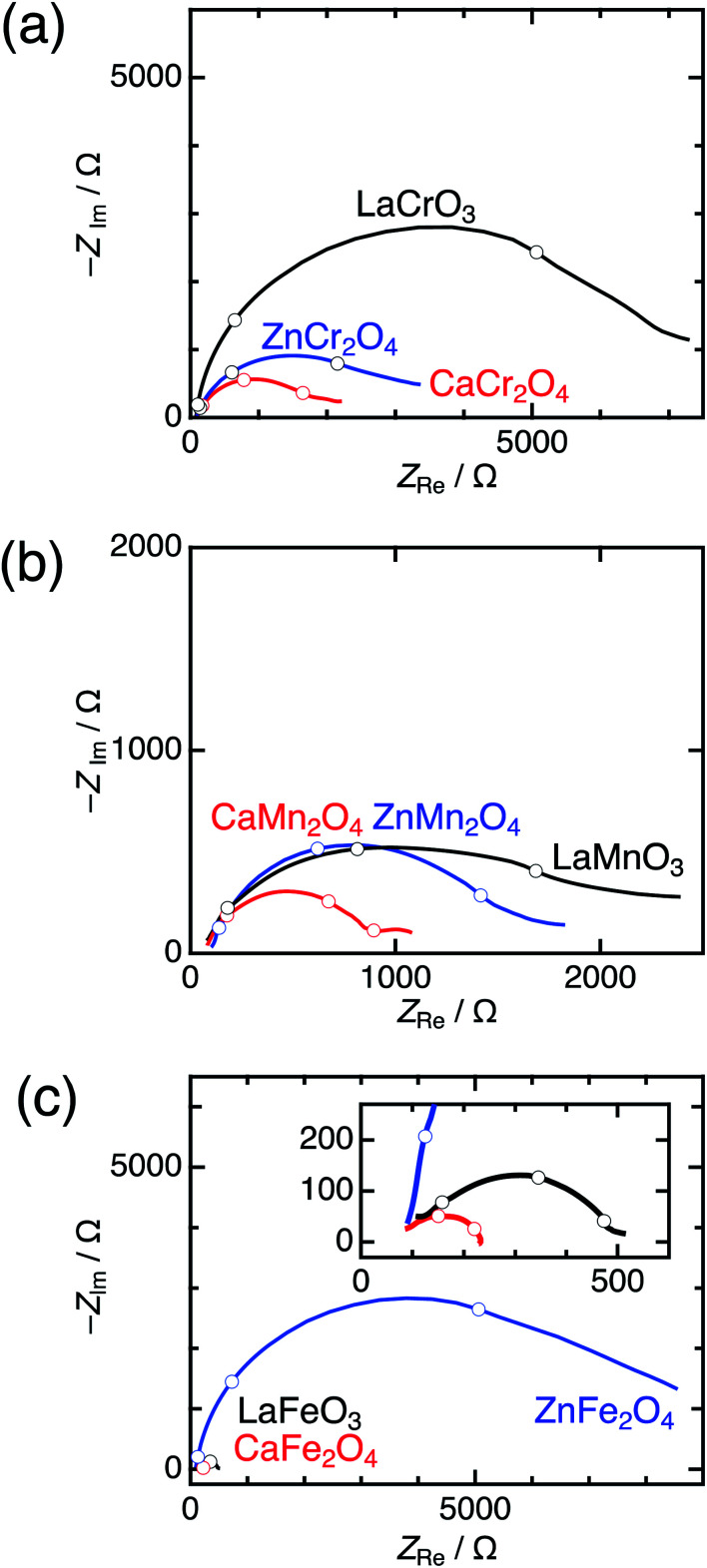
Nyquist plots of La*B*O_3_ (black), Zn*B*_2_O_4_ (blue), and Ca*B*_2_O_4_ (red) for *B* = (a) Cr, (b) Mn, and (c) Fe, measured at 1.7 V *vs.* RHE. The insets represent the magnified data in Fe oxides. Blank circles display points observed between 1–100 Hz.

Significant differences between postspinels and other structures were observed in Tafel plots and EIS analyses. Fig. 6 shows the Tafel plots for La*B*O_3_, Zn*B*_2_O_4_, and Ca*B*_2_O_4_ (*B* = Cr, Mn, and Fe). The Tafel slope of CaFe_2_O_4_ (53 mV dec^−1^) was much smaller than that of ZnFe_2_O_4_ (102 mV dec^−1^) and LaFeO_3_ (78 mV dec^−1^). Clear differences in Tafel slopes between postspinel-structured oxides and counterparts were also observed in Cr and Mn oxides. Since the Tafel slope varies in dependent on the rate-determining step (RDS),^39^ the observed differences in Tafel slope indicate that the RDS is altered by crystal structures. Nyquist plots are displayed in Fig. 7 for La*B*O_3_, Zn*B*_2_O_4_, and Ca*B*_2_O_4_ (*B* = Cr, Mn, and Fe). The semicircles around 1–100 Hz attributed to charge-transfer resistance (*R*_ct_) changed depending on the crystal structure. The *R*_ct_ value of CaFe_2_O_4_ (∼150 Ω) was smaller than that of LaFeO_3_ (∼400 Ω) and ZnFe_2_O_4_ (∼7000 Ω). CaCr_2_O_4_ (*R*_ct_ = ∼2000 Ω) and CaMn_2_O_4_ (∼800 Ω) exhibited lower *R*_ct_ values than La*B*O_3_ and Zn*B*_2_O_4_ counterparts. The fact that the *R*_ct_ values in CaCr_2_O_4_ and CaMn_2_O_4_ were larger than that in CaFe_2_O_4_ is probably associated with surface amorphizations in the formers, as shown in the HRTEM study later. The surface amorphizations disturb the charge transfer, deviating from the intrinsic nature of the crystalline surface. Consequently, the activated charge-transfer kinetics is consistently a primary origin for the enhanced OER activity in Ca*B*_2_O_4_ for Cr, Mn, and Fe oxides.

**Fig. 8:**
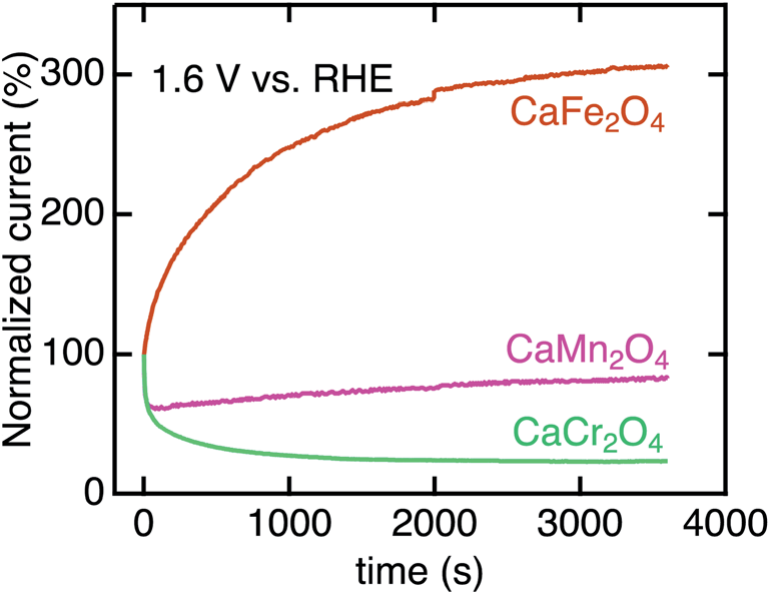
Chronoamperometry of Ca*B*_2_O_4_ for *B* = Cr, Mn, and Fe normalized initial current values. The data were collected at 1.6 V *vs.* RHE after three CV scans.

We investigated the long-term stability and surface crystalline states of Ca*B*_2_O_4_. Fig. 8 shows the CA currents normalized by initial currents in CaCr_2_O_4_, CaMn_2_O_4_, and CaFe_2_O_4_. CaFe_2_O_4_ exhibited no substantial degradation in OER activity. This observation manifests the robustness of CaFe_2_O_4_ in OER conditions, whereas the sudden drops in initial states for CaCr_2_O_4_ and CaMn_2_O_4_ (Fig. 8) indicate instability of surface crystalline states. Fig. S3† displays HRTEM images of CaCr_2_O_4_, CaMn_2_O_4_, and CaFe_2_O_4_. CaFe_2_O_4_ retained the crystalline surface after CA, as well as the pristine and as-cast powders. In contrast, CaCr_2_O_4_ demonstrated severe surface amorphization even in the as-cast sample and further evolution of the amorphous layer after CA (Fig. 8). CaMn_2_O_4_ also possessed the amorphous surface in the as-cast sample, which is probably the cause of the initial degradation in CA. Gradual increases in current density were observed for several oxides (CaMn_2_O_4_ and CaFe_2_O_4_), but the origin was unclear at the present stage. Since CaFe_2_O_4_ did not exhibit severe amorphization, the intrinsic feature of the crystalline surface is predominantly reflected in the electrochemical analyses.

Our electrochemical experiments elucidated that the OER catalytic activities in postspinels Ca*B*_2_O_4_ (*B* = Cr, Mn, and Fe) are superior to those of perovskites La*B*O_3_ and spinels Zn*B*_2_O_4_, irrespective of *B* metal ions. The commonly observed properties in Ca*B*_2_O_4_ suggest that the edge-sharing one-dimensional octahedra in postspinel structures predominate the reaction mechanism. We conducted DFT calculations to discuss the reaction mechanism on the surface of Ca*B*_2_O_4_ associated with the geometric feature of the coordination polyhedra, in addition to the bulk electronic factors possibly affecting the OER catalysis. Fig. S4† shows the DOS generated from bulk-model DFT calculations for spinels and postspinels. All Zn*B*_2_O_4_ and Ca*B*_2_O_4_ (*B* = Cr, Mn, Fe) displayed insulating band structures with band gaps of ∼1–2 eV, finding no definite difference between spinel and postspinel structures. Typical OER descriptors of O-2p band center^40^ and charge-transfer energy (Δ)^41^ were close between Zn*B*_2_O_4_ and Ca*B*_2_O_4_ (Table S10†). Unlike the linear tendency in the *η*_0.05_*vs.* Δ plots for the perovskite oxides,^32^ there was no clear trend (Fig. S5†) in the spinels and postspinels in this study. Hence, almost isoelectronic states in the bulk form cannot explain the origin of OER activity in the postspinel-structured oxides.

**Fig. 9:**
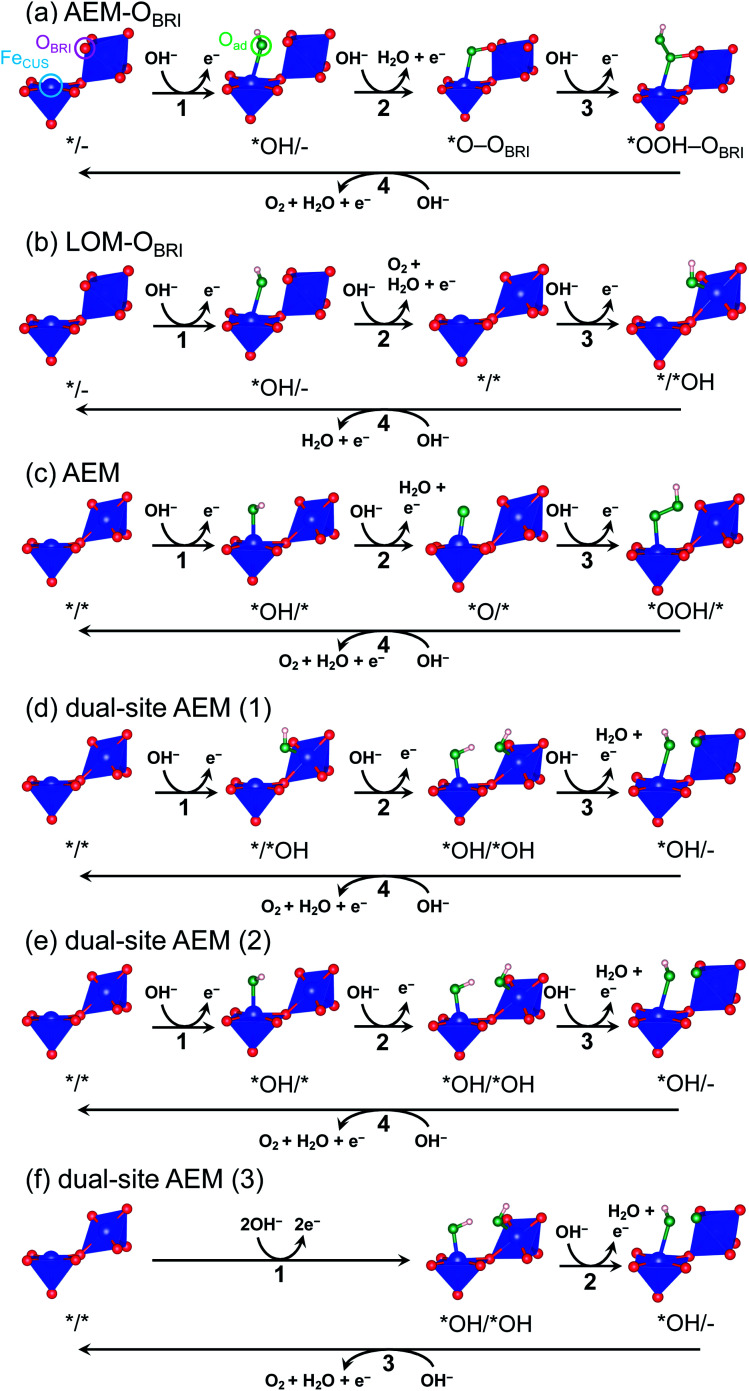
Schematics of surface structures of CaFe_2_O_4_ after structural relaxations for mechanisms of (a) AEM–O_BRI_, (b) LOM–O_BRI_, (c) AEM, and dual-site AEMs. In dual-site AEM, calculated mechanisms start from the surface of (d) */*OH, (e) *OH/*, and (f) *OH/*OH.

To evaluate credible information on the OER on postspinel, we calculated the theoretical overpotentials from the surface free-energies. In the surface-state calculations, CaFe_2_O_4_ was selected because of its robust crystalline surface in OER conditions, as demonstrated in CA and HRTEM studies. The calculations of surface states were conducted on the (001) plane with O_BRI_ ions bridged by two Fe ions, referring to the reaction mechanism in RuO_2_ with one-dimensionally aligned octahedra.^4,5^ We originally investigated three different reaction mechanisms AEM–O_BRI_, LOM–O_BRI_, and AEM. Fig. 9 shows the surface geometries after structural relaxations. First, in the AEM–O_BRI_ (Fig. 9a), the calculations were performed using conventional adsorbates, *OH, *O, and *OOH binding to Fe_CUS_ on the surface containing O_BRI_. The structural relaxation revealed the formation of additional bonds between adsorbed oxygen (O_ad_) and O_BRI_ when *O and *OOH species are adsorbed (*O–O_BRI_ and *OOH–O_BRI_ states). These bond formations indicate that O_BRI_ participates in the OER on the CaFe_2_O_4_ surface. Second, we examined the lattice oxygen mechanism^42^ (LOM–O_BRI_, Fig. 9b) in which OH^−^ is adsorbed to Fe_CUS_ (step 1: */– + OH^−^ → *OH/– + e^−^), and desorbed with O_BRI_ to evolve O_2_ (step 2: *OH/– + OH^−^ → */* + O_2_ + H_2_O + e^−^). Third, we considered the AEM mechanism, where the adsorbates are solely bound to Fe_CUS_ because of the absence of the O_BRI_ atom (Fig. 9c). This mechanism is similar to the conventional OER mechanism in single-site adsorption.

**Fig. 10:**
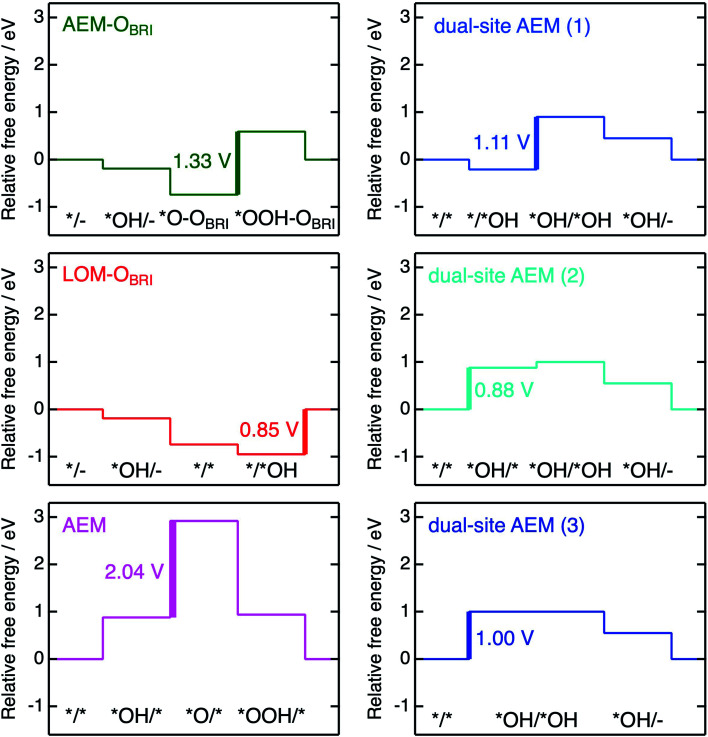
Energy diagrams for the mechanisms of AEM–O_BRI_, LOM–O_BRI_, AEM (left), and three types of dual-site AEMs (right). Thick lines and values correspond to PDSs and *η*_th_, respectively. Each relative free energy was calculated in *φ* = 1.23 V.

The free energies ΔG_*X/*Y_ at each reaction step of the above-examined models are listed in Table S17† defined with formulae in Table S14.† The value of ΔG_*X/*Y_ tended to increase in correspondence with the number of adsorbed atoms in the *X/*Y surface state. We calculated the values of energy change Δ*G*_*n*_ of each reaction steps and *η*_th_ for the six reaction mechanisms (Table S18†) as following calculations in formulae in Tables S15 and S16.†Fig. 10 shows the energy diagrams for each reaction mechanism. In Fig. 10, the thick lines represent the potential determining steps (PDSs) with the largest Δ*G*_*n*_ in each mechanism, accompanied by *η*_th_ values calculated from Δ*G*_*n*_ at PDSs. The PDSs for AEM–O_BRI_ (*η*_th_ = 1.33 V) and LOM–O_BRI_ (*η*_th_ = 0.85 V) were assigned to step 3 (*O–O_BRI_ + OH^−^ → *OOH–O_BRI_ + e^−^) and step 4 (*/*OH + OH^−^ → */– + H_2_O + e^−^), respectively. In contrast, step 2 with a significant large *η*_th_ (2.04 V) was the PDS in the AEM (*OH/* + OH^−^ → *O/* + H_2_O + e^−^). LOM–O_BRI_ demonstrated the lowest *η*_th_ among the 4-step mechanisms. Due to the high variation in surface structures and types of adsorbed species in PDSs, we could not identify any consistency in adsorption states of PDSs among the three reaction mechanisms. The PDSs of LOM–O_BRI_ and AEM were categorized as transforming steps from *OH to *O, whereas the PDSs of AEM–O_BRI_ were assigned to adsorption processes of the reactant OH^−^. We conclude that the LOM–O_BRI_ with the lowest *η*_th_ (0.85 V) examined in the present DFT calculation is the most probable mechanism to explain the high OER activity of postspinel-structured oxides.

We compared theoretical overpotential in the mechanisms reported in previous studies with LOM–O_BRI_, which is the most probable reaction mechanism among our reaction mechanisms described above. We validated two types of 4-step mechanisms, dual-site AEM (1)–(2) in Fig. 9d and e, which are reformulations of the 3-step model, and dual-site AEM (3) (Fig. 9f), proposed by Sugawara *et al.*^17^ The OH^−^ adsorbates are sequentially bonded with two Fe_CUS_ sites in the 4-step reaction with two procedures [*/* → *OH/* → *OH/*OH → *OH/– (Fig. 9d) or */* → */*OH → *OH/*OH → *OH/– (Fig. 9e)], in contrast to the simultaneous OH^−^ adsorption in the previous 3-step reaction in Fig. 9f (*/* → *OH/*OH → *OH/*O). In dual-site AEMs, depending on the initial position of *OH species, the PDSs were determined at adsorption of step 2 in dual-site AEM (1) (*η*_th_ = 1.11 V, */*OH + OH^−^ → *OH/*OH + e^−^), and step 1 in dual-site AEM (2) (*η*_th_ = 0.88 V, */* + OH^−^ → *OH/* + e^−^). The calculation using the 3-step mechanism “dual-site AEM (3)” referred to the previous study^17^ demonstrated simultaneous adsorptions of reactants (step 1) was assigned to the PDS (*η*_th_ = 1.00 V, */* + 2OH^−^ → *OH/*OH + 2e^−^). The present calculations revealed that the PDSs in three subpaths of dual-site AEMs are assigned in adsorptions steps of OH^−^ species to the surface, differing from the charge-transfer PDS in the previous study,^17^ whereas the original PDS reaction (*OH/*OH + OH^−^ → *OH/– + H_2_O + e^−^) was stabilized in our calculations. Since the repulsive energies between the two OH^−^ species were not considered, the theoretical overpotential (0.58 V)^17^ in the previous 3-step reaction mechanism would be underestimated.

## Conclusion

In summary, we investigated the OER catalytic activity in the postspinel-structured oxides Ca*B*_2_O_4_ (*B* = Cr, Mn, and Fe), revealing higher OER activities and smaller charge-transfer resistances than the perovskite- and spinel-structured counterparts. The DFT calculation on the surface of CaFe_2_O_4_ elucidates that a novel reaction mechanism with the lowest theoretical overpotential, where O_BRI_ and O_ad_ are combined to generate oxygen, is more probable than the 3-step reaction mechanism with simultaneous adsorption of OH^−^ proposed in the previous study. Consequently, the geometric configurations around adsorption sites tolerating additional bonding are another factor to activate OER beyond the conventional single-site OER mechanism.

## Conflicts of interest

There are no conflicts to declare.

## Supplementary Material

RA-012-D2RA00448H-s001
