# Supplementary material for: Highly active postspinel-structured catalysts for oxygen evolution reaction
Source: RSC Adv. 2022 Feb 10;12(9):5094–104. doi: 10.1039/d2ra00448h (PMC8981248; doi:10.1039/d2ra00448h)
Supplement: RA-012-D2RA00448H-s001 [file RA-012-D2RA00448H-s001.pdf]

Electronic Supplementary Material (ESI) for RSC Advances.
This journal is © The Royal Society of Chemistry 2022

Electronic Supplementary Information

Highly active postspinel-structured catalysts for oxygen evolution reaction

Yuichi Okazaki,[a] Seiji Oda,[a] Akihiko Takamatsu,[b] Shogo Kawaguchi,[c] Hirofumi Tsukasaki,[a] Shigeo Mori,[a] Shunsuke Yagi,[d] Hidekazu Ikeno,[ae]* Ikuya Yamada[a]*

*[a] Department of Materials Science, Graduate School of Engineering, Osaka Prefecture University, Sakai 599-8570, Japan. *E-mail: yamada@mtr.osakafu-u.ac.jp (I.Y.), ikeno@mtr.osakafu-u.ac.jp (H.I.)*

[b] Department of Molecular Engineering, Graduate School of Engineering, Kyoto University, Kyoto 615-8510, Japan

[c] Japan Synchrotron Radiation Research Institute (JASRI), 679-5198.

[d] Institute of Industrial Science, The University of Tokyo, Meguro-ku 153-8505.

[e] Precursory Research for Embryonic Science and Technology (PRESTO), Japan Science and Technology Agency (JST), Kawaguchi 332-0012, Japan

Table S1 Structure parameters and BVSs for $CaCr_2O_4$ obtained from the Rietveld refinement.

Atom	Site	x	y	z	$U_{iso}\times$ 1000 (Å^2)	BVS
Ca	4*c*	0.75957(9)	1/4	0.65902(7)	4.26(17)	2.41
Cr1	4*c*	0.43999(6)	1/4	0.61270(5)	1.73(10)	2.78
Cr2	4*c*	0.41686(6)	1/4	0.10092(5)	1.73(10)	2.77
O1	4*c*	0.2048(3)	1/4	0.1581(2)	4.2(3)	–1.82
O2	4*c*	0.1168(2)	1/4	0.4742(2)	4.2(3)	–2.11
O3	4*c*	0.5249(3)	1/4	0.7839(2)	4.2(3)	–1.94
O4	4*c*	0.4172(2)	1/4	0.4271(2)	4.2(3)	–2.11

Space group: *Pnma* (No. 62); a = 9.08312(6) Å, b = 2.96848(2) Å, c = 10.62420(7) Å; R_{wp} = 6.514%, R_B = 3.056%, S = 1.2871. The occupancy factors for all atoms were fixed to the unity. The BVSs were calculated using the following parameters: b_0 = 0.37 for all atoms, r_0 = 1.967 for Ca^{2+} and r_0 = 1.724 for Cr^{3+}.

Table S2 Structure parameters and BVSs for $CaMn_2O_4$ obtained from the Rietveld refinement.

Atom	Site	x	y	z	$U_{iso}\times$ 1000 (Å^2)	BVS
Ca	4*d*	0.6816(4)	0.35085(10)	1/4	5.5(3)	2.25
Mn	8*e*	0.20386(17)	0.11167(5)	0.06853(5)	3.95(15)	2.97
O1	4*c*	0.5934(9)	1/4	0	6.3(4)	–2.10
O2	4*d*	0.1935(10)	0.1835(3)	1/4	6.3(4)	–2.21
O3	8*e*	0.2022(7)	0.4751(2)	0.1062(2)	6.3(4)	–2.11

Space group: *Pbcm* (No. 57); a = 3.15881(2) Å, b = 9.99484(8) Å, c = 9.67905(8) Å; R_{wp} = 8.723%, R_B = 4.211%, S = 2.1367. The occupancy factors for all atoms were fixed to the unity. The BVSs were calculated using the following parameters: b_0 = 0.37 for all atoms, r_0 = 1.967 for Ca^{2+} and r_0 = 1.760 for Mn^{3+}.

Table S3 Structure parameters and BVSs for $CaFe_2O_4$ obtained from the Rietveld refinement.

Atom	Site	*x*	*y*	*z*	$U_{iso}\times 1000$ (Å^2)	BVS
Ca	4*c*	0.24365(9)	1/4	0.34597(7)	4.76(16)	2.23
Fe1	4*c*	0.06640(6)	1/4	0.11190(5)	3.15(9)	2.79
Fe2	4*c*	0.08135(6)	1/4	0.60528(5)	3.15(9)	2.84
O1	4*c*	0.2927(2)	1/4	0.6628(2)	3.4(3)	–1.81
O2	4*c*	0.3821(2)	1/4	0.9768(2)	3.4(3)	–2.02
O3	4*c*	0.4762(3)	1/4	0.2173(2)	3.4(3)	–1.96
O4	4*c*	0.0803(2)	1/4	0.92715(19)	3.4(3)	–2.06

Space group: *Pnma* (No. 62); a = 9.22725(7) Å, b = 3.02020(2) Å, c = 10.69728(8) Å; R_{wp} = 5.178%, R_B = 2.276%, S = 1.7666. The occupancy factors for all atoms were fixed to the unity. The BVSs were calculated using the following parameters: b_0 = 0.37 for all atoms, r_0 = 1.967 for Ca^{2+} and r_0 = 1.759 for Fe^{3+}.

Table S4 Structure parameters and BVS for $ZnCr_2O_4$ obtained from the Rietveld refinement.

Atom	Site	*x*	*y*	*z*	$U_{iso}\times1000$ (Å^2)	BVS
Zn	8*a*	1/8	1/8	1/8	4.10(5)	1.91
Cr	16*d*	1/2	1/2	1/2	2.22(5)	2.95
O	32*e*	0.26205(5)	0.26205(5)	0.26205(5)	2.49(15)	–1.95

Space group: $Fd\bar{3}m$ (No. 227); a = 8.32708(2) Å; R_{wp} = 4.422%, R_B = 1.820%, S = 1.8272; The occupancy factors for all atoms were fixed to the unity. The BVSs were calculated using the following parameters: b_0 = 0.37 for all atoms, r_0 = 1.704 for Zn^{2+} and r_0 = 1.724 for Cr^{3+}.

Table S5 Structure parameters and BVSs for $ZnMn_2O_4$ obtained from the Rietveld refinement.

Atom	Site	*x*	*y*	*z*	U_{iso}×1000 (Å^2)	BVS
Zn	4*b*	0	$^1/_4$	$^3/_8$	5.30(13)	1.85
Mn	8*c*	0	0	0	3.69(12)	3.11
O	16*h*	0	0.4743(3)	0.25627(17)	4.7(3)	–1.97

Space group: $I4_1/amd$ (No. 141); a = 5.71745(2) Å; c = 9.24605(4) Å; R_{wp} = 9.47%, R_B = 4.14%, S = 2.57. The occupancy factors for all atoms were fixed to the unity. The BVSs were calculated using the following parameters: b_0 = 0.37 for all atoms, r_0 = 1.704 for Zn^{2+} and r_0 = 1.760 for Mn^{3+}.

Table S6 Structure parameters and BVS for $ZnFe_2O_4$ obtained from the Rietveld refinement.

Atom	Site	*x*	*y*	*z*	U_{iso}×1000 (Å^2)	BVS
Zn	8*a*	$^1/_8$	$^1/_8$	$^1/_8$	6.93(11)	1.85
Fe	16*d*	$^1/_2$	$^1/_2$	$^1/_2$	5.06(9)	2.95
O	32*e*	0.26106(12)	0.26106(12)	0.26106(12)	6.2(3)	–1.88

Space group: $Fd\bar{3}m$ (No. 227); a = 8.44198(4) Å; R_{wp} = 0.729%, R_B = 3.632%, S = 1.8178. The occupancy factors for all atoms were fixed to the unity. The BVSs were calculated using the following parameters: b_0 = 0.37 for all atoms, r_0 = 1.704 for Zn^{2+} and r_0 = 1.759 for Fe^{3+}.

Table S7 Structure parameters and BVS for $LaCrO_3$ obtained from the Rietveld refinement.

Atom	Site	*x*	*y*	*z*	U_{iso}×1000 (Å^2)	BVS
La	4*c*	0.01892(5)	$^1/_4$	0.00576(8)	2.39(7)	2.549
Cr	*4b*	0	0	$^1/_2$	1.25(10)	3.127
O1	4*c*	0.4918(6)	$^1/_4$	0.9501(8)	11.1(6)	–0.553
O2	8*d*	0.2712 (8)	0.0323(4)	0.7266(7)	11.1(6)	–0.546

Space group: *Pnma* (No.62); a = 5.48026(6) Å, b = 7.76122(8) Å, c = 5.51639(6) Å; R_{wp} = 5.77 %, R_B = 1.58 %, S = 1.26. The occupancy factors for all atoms were fixed to the unity. The BVSs were calculated using the following parameters: b_0 = 0.37 for all atoms, r_0 = 2.172 for La^{3+} and r_0 = 1.724 for Cr^{3+}.

Table S8 Structure parameters and BVS for $LaMnO_3$ obtained from the Rietveld refinement.

Atom	Site	*x*	*y*	*z*	U_{iso}×1000 (Å^2)	BVS
La	6*a*	0	0	¼	10.21(10)	2.580
Mn	6*b*	0	0	0	4.89(15)	3.468
O	18*e*	0.4487(4)	0	¼	8.1(6)	–0.553

Space group: $R\bar{3}c$ (No. 167); a = 5.52164(9) Å, c = 13.31879(16) Å; R_{wp} = 7.48%, R_B = 2.30 %, S = 1.8912. The occupancy factors for all atoms were fixed to the unity. The BVSs were calculated using the following parameters: b_0 = 0.37 for all atoms, r_0 = 2.172 for La^{3+} and r_0 = 1.760 for Mn^{3+}.

Table S9 Structure parameters and BVS for $LaFeO_3$ obtained from the Rietveld refinement.

Atom	Site	*x*	*y*	*z*	U_{iso}×1000 (Å^2)	BVS
La	4*c*	0.02966(6)	¼	0.9936(9)	2.77(8)	2.72
Fe	*4b*	0	0	½	1.82(13)	3.07
O1	4c	0.4863(8)	¼	0.0748(11)	7.0(6)	–0.51
O2	32*e*	0.26106(12)	0.26106(12)	0.26106(12)	7.0(6)	–0.51

Space group: *Pnma* (No.62); a = 5.56698(4) Å, b = 7.85474(6) Å, c = 5.55458(4) Å; R_{wp} = 8.61 %, R_B = 3.40 %, S = 2.19. The occupancy factors for all atoms were fixed to the unity. The BVSs were calculated using the following parameters: b_0 = 0.37 for all atoms, r_0 = 2.172 for La^{3+} and r_0 = 1.759 for Fe^{3+}.

Table S10 The 2p band center of oxygen (ε_{2p}), unoccupied 3d band center of transition metal ($\varepsilon_{3d\text{-}un}$), and charge-transfer energies(Δ) for ZnB_2O_4 and CaB_2O_4 (B = Cr, Mn, Fe).

Compound	ε_{2p} (eV)	$\varepsilon_{3d\text{-}un}$ (eV)	Δ (eV)
$ZnCr_2O_4$	−3.08	4.68	7.76
$ZnMn_2O_4$	−3.69	3.49	7.18
$ZnFe_2O_4$	−2.95	2.52	5.48
$CaCr_2O_4$	−2.93	4.44	7.38
$CaMn_2O_4$	−2.65	4.14	6.79
$CaFe_2O_4$	−2.58	2.69	5.26

Table S11 Magnetic structures and nominal electron configurations for ZnB_2O_4 and CaB_2O_4 (B = Cr, Mn, and Fe).

Compound	Magnetic structure*	Nominal electron configuration
$ZnCr_2O_4$	FM	$t_{2g}^3e_g^0$
$ZnMn_2O_4$	FM	$t_{2g}^3e_g^1$
$ZnFe_2O_4$	FM	$t_{2g}^3e_g^2$
$CaCr_2O_4$	AFM	$t_{2g}^3e_g^0$
$CaMn_2O_4$	AFM	$t_{2g}^3e_g^1$
$CaFe_2O_4$	AFM	$t_{2g}^3e_g^2$

*FM: ferromagnetic, AFM: antiferromagnetic.

Table S12 Reaction paths for AEM-O_{BRI}, LOM-O_{BRI}, and AEM.

Reaction step n	AEM-O_{BRI}	LOM-O_{BRI}	AEM
1	*/- + H_2O ⇋ *OH/- + H^+ + e^-	*/- + H_2O ⇋ *OH/- + H^+ + e^-	*/* + H_2O ⇋ *OH/* + H^+ + e^-
2	*OH/- ⇋ *O–O_{BRI} + H^+ + e^-	*OH/- ⇋ */* + O_2(g) + H^+ + e^-	*OH/* ⇋ *O/* + H^+ + e^-
3	*O/- + H_2O ⇋ *OOH–O_{BRI} + H^+ + e^-	*/* + H_2O ⇋ */*OH + H^+ + e^-	*O/* + H_2O ⇋ *OOH/* + H^+ + e^-
4	*OOH–O_{BRI} ⇋ */- + O_2(g) + H^+ + e^-	*/*OH ⇋ */- + H^+ + e^-	*OOH/* ⇋ */* + O_2(g) + H^+ + e^-

Table S13 Reaction paths for three types of dual-site AEMs

Reaction step n	dual-site AEM (1)	dual-site AEM (2)	dual-site AEM (3)
1	*/* + H_2O ⇋ */*OH + H^+ + e^-	*/* + H_2O ⇋ *OH/* + H^+ + e^-	*/* + 2H_2O ⇋ *OH/*OH + 2H^+ + 2e^-
2	*/*OH + H_2O ⇋ *OH/*OH + H^+ + e^-	*OH/* + H_2O ⇋ *OH/*OH + H^+ + e^-	*OH/*OH ⇋ *OH/- + H^+ + e^-
3	*OH/*OH ⇋ *OH/- + H^+ + e^-	*OH/*OH ⇋ *OH/- + H^+ + e^-	*OH/- ⇋ */* + O_2(g) + H^+ + e^-
4	*OH/- ⇋ */* + O_2(g) + H^+ + e^-	*OH/- ⇋ */* + O_2(g) + H^+ + e^-	

Table S14 Change of free energy $\Delta G_{*X/*Y}$ for $*X/*Y$ surface states.

Surface state	$\Delta G_{*X/*Y}$
*OH/-	$\Delta G_{*\mathrm{OH/-}} = E_{*\mathrm{OH/-}} - E_{*} - \left(2E_{\mathrm{H_2O}} - \frac{3}{2}E_{\mathrm{H_2}}\right) + [\Delta\mathrm{ZPE} - T\Delta S]_{*\mathrm{OH/*}}$
*O–O$_{BRI}$	$\Delta G_{*\mathrm{O\text{-}O_{BRI}}} = E_{*\mathrm{O/–}} - E_{*} - \left(2E_{\mathrm{H_2O}} - 2E_{\mathrm{H_2}}\right) + [\Delta\mathrm{ZPE} - T\Delta S]_{*\mathrm{O\text{-}O_{BRI}}}$
*OOH–O$_{BRI}$	$\Delta G_{*\mathrm{OOH\text{-}O_{BRI}}} = E_{*\mathrm{OOH/–}} - E_{*} - \left(3E_{\mathrm{H_2O}} - \frac{5}{2}E_{\mathrm{H_2}}\right) + [\Delta\mathrm{ZPE} - T\Delta S]_{*\mathrm{OOH\text{-}}}$ [illegible]
/	$\Delta G_{*/*} = E_{*\mathrm{OH/*}} - E_{*} - \left(E_{\mathrm{H_2O}} - \frac{1}{2}E_{\mathrm{H_2}}\right) + [\Delta\mathrm{ZPE} - T\Delta S]_{*/*}$
*/*OH	$\Delta G_{*/*\mathrm{OH}} = E_{*/*\mathrm{OH}} - E_{*} - \left(E_{\mathrm{H_2O}} - \frac{1}{2}E_{\mathrm{H_2}}\right) + [\Delta\mathrm{ZPE} - T\Delta S]_{*/*\mathrm{OH}}$
OH/	$\Delta G_{*\mathrm{OH/*}} = E_{*\mathrm{OH/*}} - E_{*} - \left(E_{\mathrm{H_2O}} - \frac{1}{2}E_{\mathrm{H_2}}\right) + [\Delta\mathrm{ZPE} - T\Delta S]_{*\mathrm{OH/*}}$
O/	$\Delta G_{*\mathrm{O/*}} = E_{*\mathrm{O/*}} - E_{*} - \left(E_{\mathrm{H_2O}} - E_{\mathrm{H_2}}\right) + [\Delta\mathrm{ZPE} - T\Delta S]_{*\mathrm{O/*}}$
OOH/	$\Delta G_{*\mathrm{OOH/*}} = E_{*\mathrm{OOH/*}} - E_{*} - \left(2E_{\mathrm{H_2O}} - \frac{3}{2}E_{\mathrm{H_2}}\right) + [\Delta\mathrm{ZPE} - T\Delta S]_{*\mathrm{OOH/*}}$
*OH/*OH	$\Delta G_{*\mathrm{OH/*OH}} = E_{*\mathrm{OH/*OH}} - E_{*} - \left(2E_{\mathrm{H_2O}} - E_{\mathrm{H_2}}\right) + 2[\Delta\mathrm{ZPE} - T\Delta S]_{*\mathrm{OH/*}}$ [illegible]

where $[\Delta\mathrm{ZPE} - T\Delta S]_{*X/*Y}$ is calculated from change of ZPE and S for each $*X/*Y$ surface state. E_* is the energy with the surface including O$_{BRI}$ lattice oxygen (the */- surface).

Table S15 Relative free energies ΔG_n (units: eV) for AEM-O$_{BRI}$, LOM-O$_{BRI}$, and AEM.

ΔG_n	AEM-O$_{BRI}$	LOM-O$_{BRI}$	AEM
ΔG_1	$\Delta G_{*\mathrm{OH/-}} - e\phi + k_BT\ln a_{\mathrm{H^+}}$	$\Delta G_{*\mathrm{OH/-}} - e\phi + k_BT\ln a$ [illegible]	$\Delta G_{*\mathrm{OH/*}} - \Delta G_{*/*} - e\phi +$ [illegible]
ΔG_2	$\Delta G_{*\mathrm{O\text{-}O_{BRI}}} - \Delta G_{*\mathrm{OH/-}} - e\phi$	$\Delta G_{*/*} - \Delta G_{*\mathrm{OH/-}} - e\phi$	$\Delta G_{*\mathrm{O/*}} - \Delta G_{*\mathrm{OH/*}} - e\phi$ [illegible]
ΔG_3	$\Delta G_{*\mathrm{OOH\text{-}O_{BRI}}} - \Delta G_{*\mathrm{O\text{-}O_{BRI}}}$ [illegible] $+ k_BT\ln a_{\mathrm{H^+}}$	$\Delta G_{*/*\mathrm{OH}} - \Delta G_{*/*} - e\phi$	$\Delta G_{*\mathrm{OOH/*}} - \Delta G_{*\mathrm{O/*}} - e\phi$
ΔG_4	$4.92[\mathrm{eV}] - \Delta G_{*\mathrm{OOH\text{-}O_{BRI}}} - e$ [illegible] $+ k_BT\ln a_{\mathrm{H^+}}$	$4.92[\mathrm{eV}] - \Delta G_{*/*\mathrm{OH}} - e$ [illegible] $+ k_BT\ln a_{\mathrm{H^+}}$	$4.92[\mathrm{eV}] - (\Delta G_{*\mathrm{OOH/*}} - \Delta G_{*/*}) - e\phi + k_BT\ln a$ [illegible]

Table S16 Relative free energies ΔG_n (units: eV) for dual-site AEM (1), (2), and (3).

ΔG_n	dual-site AEM (1)	dual-site AEM (2)	dual-site AEM (3)
ΔG_1	$\Delta G_{*/*\mathrm{OH}} - \Delta G_{*/*} - e\phi + k_BT\ln a_{\mathrm{H^+}}$	$\Delta G_{*\mathrm{OH/*}} - \Delta G_{*/*} - e\phi + k_BT\ln a_{\mathrm{H^+}}$	$\Delta G_{*\mathrm{OH/*OH}} - \Delta G_{*/*} - e\phi + k_BT\ln a_{\mathrm{H^+}}$
ΔG_2	$\Delta G_{*\mathrm{OH/*OH}} - \Delta G_{*/*\mathrm{OH}} - e\phi + k_BT\ln a_{\mathrm{H^+}}$	$\Delta G_{*\mathrm{OH/*OH}} - \Delta G_{*\mathrm{OH/*}} - e\phi + k_BT\ln a_{\mathrm{H^+}}$	$\Delta G_{*\mathrm{OH/-}} - \Delta G_{*\mathrm{OH/*OH}} - e\phi + k_BT\ln a_{\mathrm{H^+}}$
ΔG_3	$\Delta G_{*\mathrm{OH/-}} - \Delta G_{*\mathrm{OH/*OH}} - e\phi + k_BT\ln a_{\mathrm{H^+}}$	$\Delta G_{*\mathrm{OH/-}} - \Delta G_{*\mathrm{OH/*OH}} - e\phi + k_BT\ln a_{\mathrm{H^+}}$	$4.92 - (\Delta G_{*\mathrm{OH/-}} - \Delta G_{*/*}$ [illegible] $- e\phi + k_BT\ln a_{\mathrm{H^+}}$
ΔG_4	$4.92 - (\Delta G_{*\mathrm{OH/-}} - \Delta G_{*/*}$ [illegible]	$4.92 - (\Delta G_{*\mathrm{OH/-}} - \Delta G_{*/*}$ [illegible]	-

$- e\phi + k_B T \ln a_{H^+}$ $- e\phi + k_B T \ln a_{H^+}$

Table S17 DFT-calculated $\Delta G_{*X/*Y}$ (unit: eV) for each $*X/*Y$ surface state in (001) $CaFe_2O_4$ surface.

$\Delta G_{*OH/-}$	$\Delta G_{*O-O_{BRI}}$	$\Delta G_{*OOH-O_{BRI}}$	$\Delta G_{*/*}$	$\Delta G_{*/*OH}$	$\Delta G_{*OH/*}$	$\Delta G_{*O/*}$	$\Delta G_{*OOH/*}$	$\Delta G_{*OH/*OH}$
1.04	1.72	4.28	–3.20	–2.08	–1.09	2.18	1.43	0.26

In these calculations, the ΔG values are defined as the free energy change with respect to the */- surface where the Fe_{CUS} ion is exposed, and the O_{BRI} is bound.

Table S18 DFT-calculated ΔG_n (unit: eV) for each reaction step n, and theoretical overpotential (η_{th}) (unit: V).

Reaction step n/ η_{th}	ΔG_n					
	(a)	(b)	(c)	(d)	(e)	(f)
1	1.04	1.04	2.11	1.02	2.11	3.46
2	0.68	0.68	3.27	2.34	1.35	0.78
3	2.56	1.02	–0.75	0.78	0.78	0.76
4	0.64	2.08	0.29	0.76	0.76	-
η_{th}	1.33	0.85	2.04	1.11	0.88	1.00

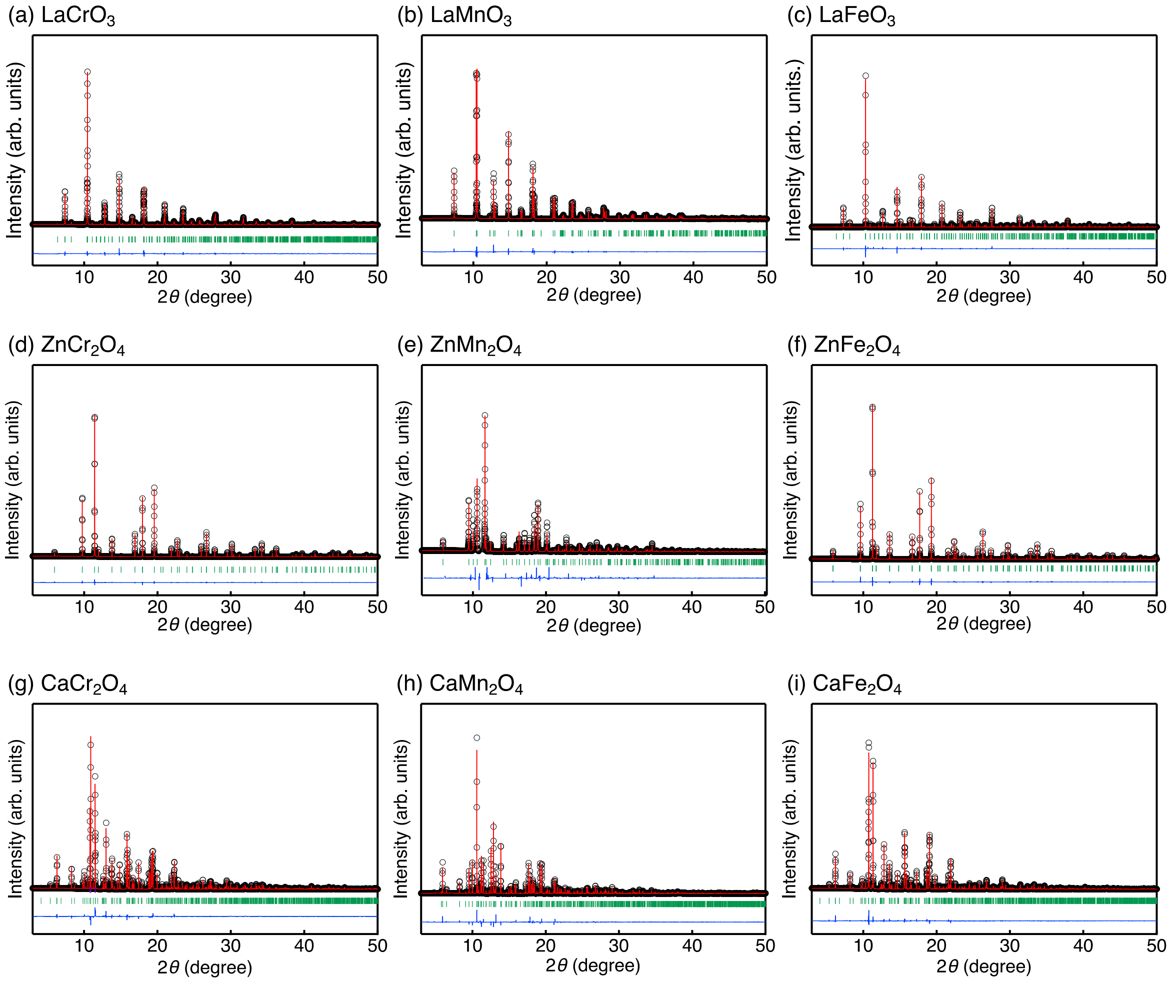

Fig. S1 Rietveld refinement result of SXRD data for (a) $LaCrO_3$, (b) $LaMnO_3$, (c) $LaFeO_3$, (d) $ZnCr_2O_4$, (e) $ZnMn_2O_4$, (f) $ZnFe_2O_4$, (g) $CaCr_2O_4$, (h) $CaMn_2O_4$, and (i) $CaFe_2O_4$. Circles (black) and solid lines (red) represent observed and calculated patterns, respectively. The difference between the observed and calculated patterns is shown at the bottom (blue). The vertical marks (green) indicate the Bragg reflection positions.

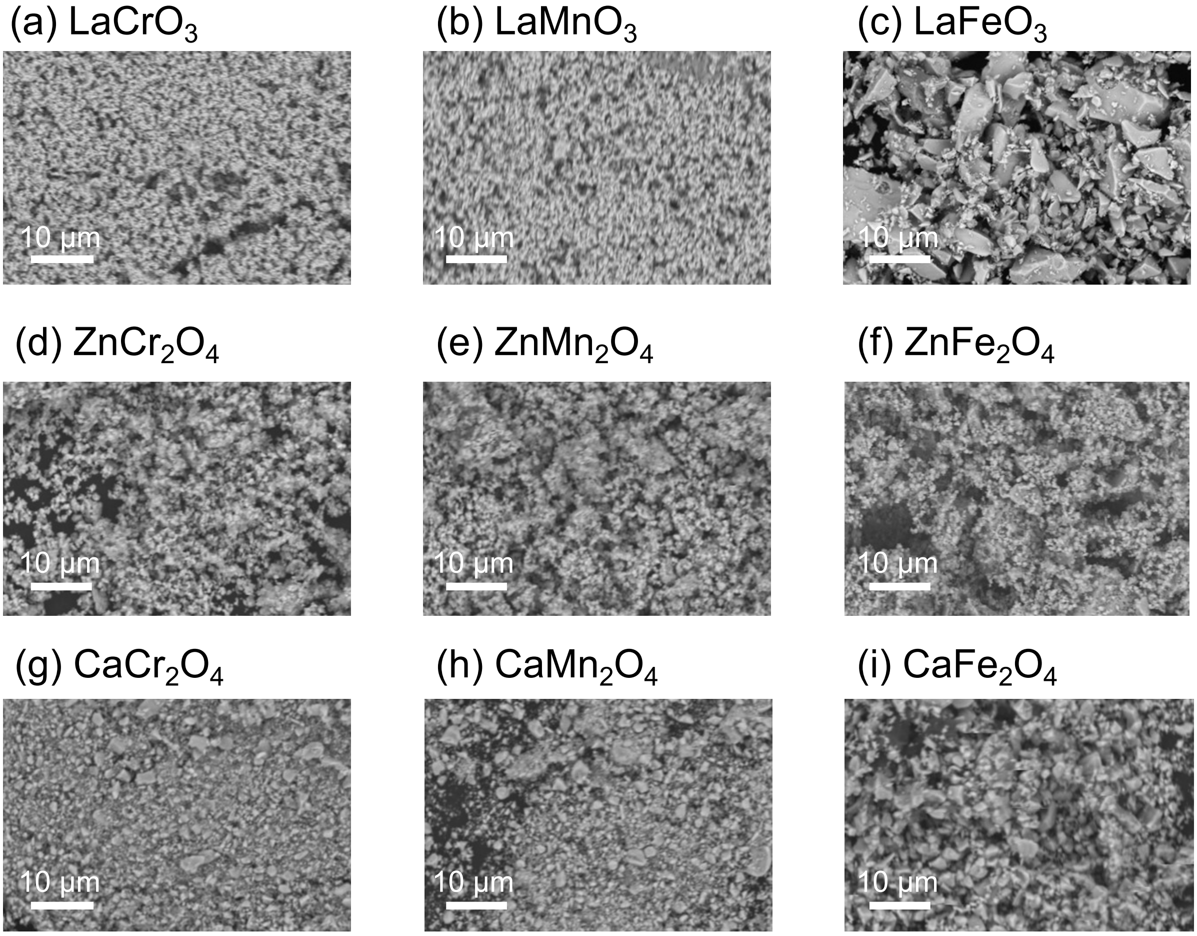

Fig. S2 SEM images for perovskite oxides: (a) $LaCrO_3$, (b) $LaMnO_3$, and (c) $LaFeO_3$, spinel oxides: (a) $ZnCr_2O_4$, (b) $ZnMn_2O_4$, and (c) $ZnFe_2O_4$, and postspinel oxides: (d) $CaCr_2O_4$, (e) $CaMn_2O_4$, and (f) $CaFe_2O_4$.

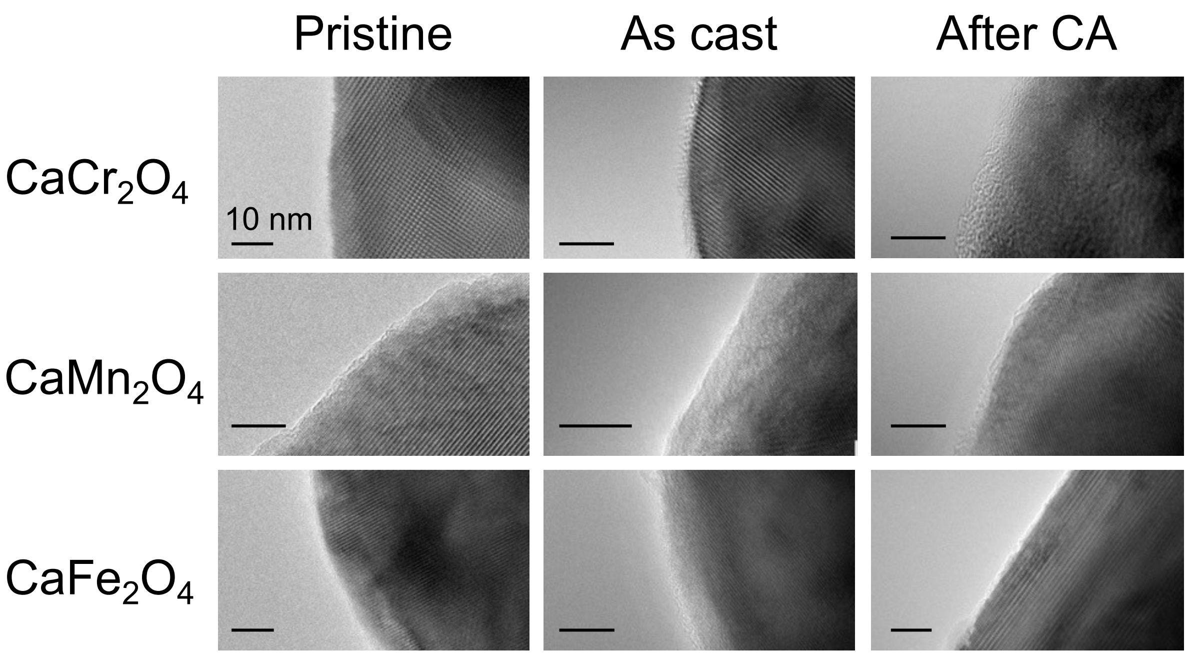

Fig. S3 HRTEM images of postspinel oxides, $CaCr_2O_4$, $CaMn_2O_4$, and $CaFe_2O_4$ in pristine (left), as-cast (middle), after chronoamperometry (right) at 1.6 V vs. RHE for 1h. The bars show 10 nm.

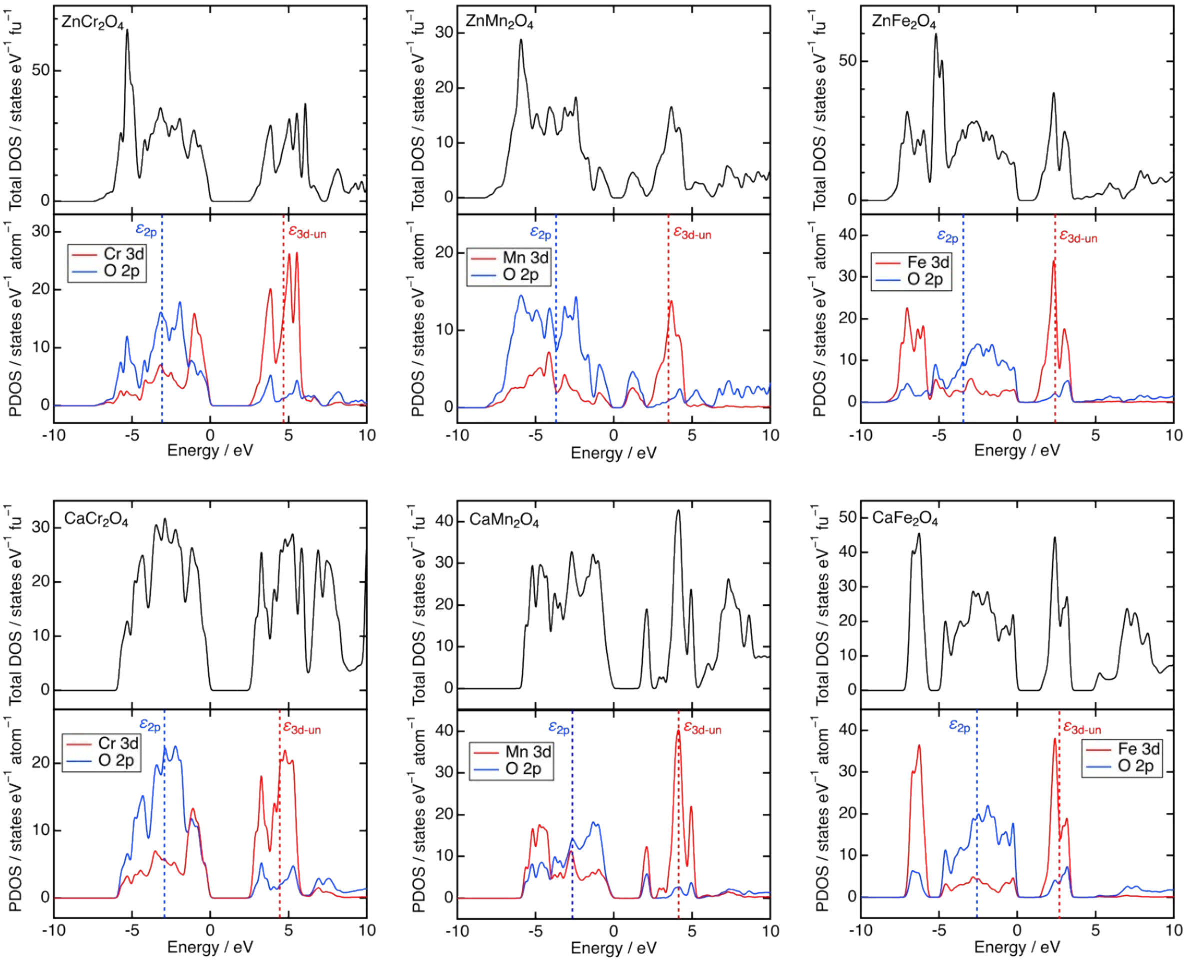

Fig. S4 Total density of states (DOS) and partial DOS (PDOS) for ZnB_2O_4 and CaB_2O_4 (B = Cr, Mn, Fe) obtained by DFT calculation.

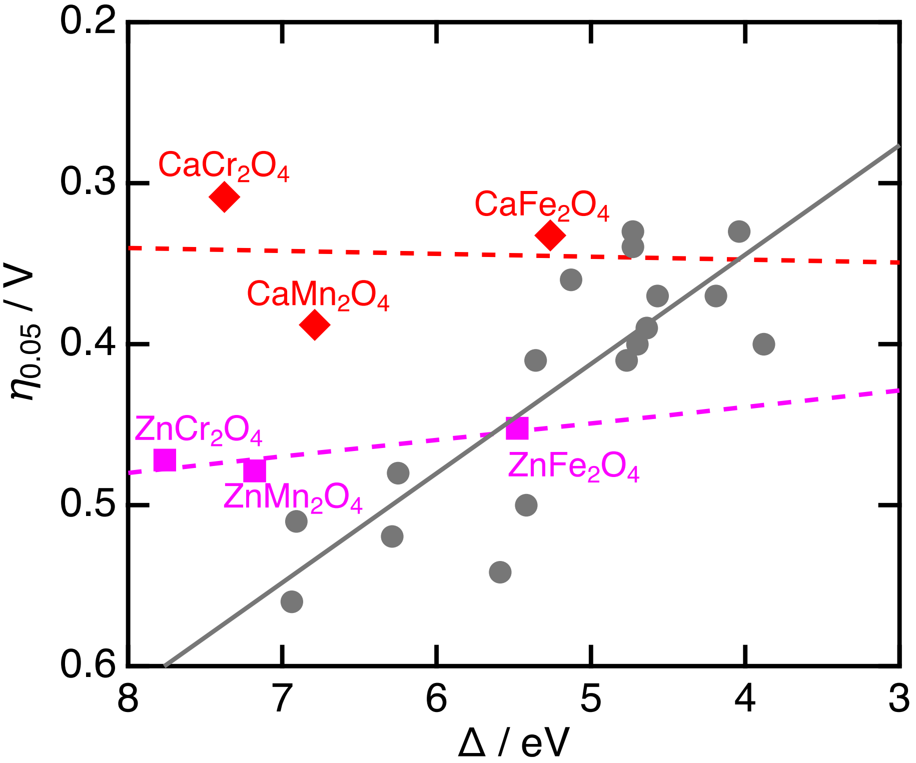

Fig. S5 Overpotential as a function of charge-transfer energy (Δ) for ZnB_2O_4, LaBO_3, and CaB_2O_4. The data for perovskite oxides were taken from the reference.[1,2] The lines were obtained from linear fitting.

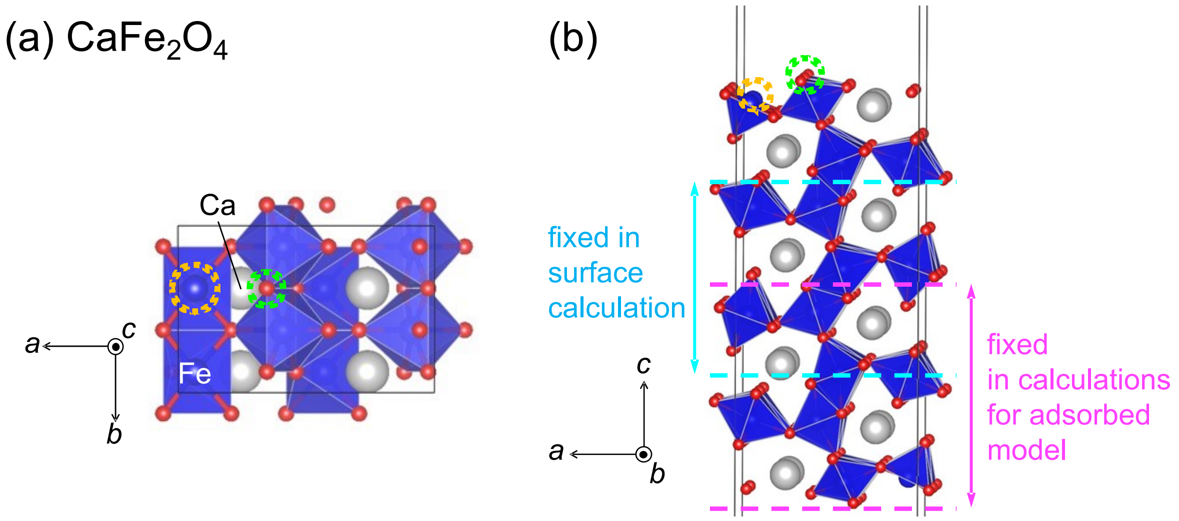

Fig. S6 Schematics for slab models for $CaFe_2O_4$ (001) plane. Top views are illustrated in (a). The Fe_{CUS} and O_{BRI} were enclosed in dashed circles (orange and green), respectively. Fixed layers in surface calculations (blue areas) and calculations for adsorbed models (magenta areas) are displayed in the side view (b) for $CaFe_2O_4$.

References

1 I. Yamada, A. Takamatsu, K. Asai, H. Ohzuku, T. Shirakawa, T. Uchimura, S. Kawaguchi, H. Tsukasaki, S. Mori, K. Wada, H. Ikeno and S. Yagi, *ACS Appl. Energy Mater.*, 2018, **1**, 3711–3721.

2 I. Yamada, A. Takamatsu, K. Asai, T. Shirakawa, H. Ohzuku, A. Seno, T. Uchimura, H. Fujii, S. Kawaguchi, K. Wada, H. Ikeno and S. Yagi, *J. Phys. Chem. C*, 2018, **122**, 27885–27892.
